# Metagenomic Insights into Gut Microbiota Alterations Following *Dendrobium huoshanense* Water Extract Intervention in Streptozotocin-Induced Type 1 Diabetic Rats

**DOI:** 10.3390/ijms27125308

**Published:** 2026-06-11

**Authors:** Hai-Jun Xu, Qing-Le Liu, Ya-Fei Zhang, Shu-Nan Cuan, Zhe Jia, Deliang Qiao

**Affiliations:** College of Life and Health, West Anhui University, Lu’an 237012, China

**Keywords:** *Dendrobium huoshanense*, type 1 diabetes, gut microbiota, metagenomics, water extract, rat

## Abstract

*Dendrobium huoshanense* water extract (DHWE) exhibits hypoglycemic effects in streptozotocin-induced type 1 diabetic (STZ-T1D) rats. However, its regulatory impact on the gut microbiota of T1D rats remains largely unclear. In this study, metagenomic sequencing was employed to characterize alterations in the gut microbiota of STZ-T1D rats following DHWE intervention, aiming to explore associations between DHWE-mediated gut microbial changes and T1D-related phenotypes. The results showed that 1300 mg/kg·BW/day DHWE did not significantly affect gut microbial α-diversity (*p* > 0.05), but drove the β-diversity structure toward that of normal rats. Meanwhile, DHWE significantly reduced the Bacteroidota/Bacillota ratio (*p* < 0.05), *Megamonas* (*p* < 0.01), *Megamonas funiformis* (*p* < 0.01), and notably increased the relative abundances of *Adlercreutzia* (*p* < 0.01), *Adlercreutzia equolifaciens* (*p* < 0.01) in STZ-T1D rats. Furthermore, functional annotation revealed that DHWE enriched multiple metabolic pathways, including streptomycin biosynthesis, ansamycins biosynthesis, galactose metabolism, ether lipid metabolism, and caprolactam degradation. Collectively, these findings demonstrate that DHWE reshapes gut microbiota composition and function in STZ-T1D rats, offering new clues regarding how gut microbial changes may contribute to the modulatory effects of *Dendrobium huoshanense* in T1D conditions.

## 1. Introduction

Type 1 diabetes (T1D) is a chronic metabolic disorder characterized by absolute insulin deficiency resulting from immune-mediated destruction of pancreatic islet β-cells [1]. Its incidence has been increasing globally [2]. The clinical management of T1D remains challenging, requiring rigorous monitoring and interventions to prevent acute metabolic disturbances and long-term vascular complications. Current standard therapy depends on exogenous insulin delivered by injection or pump, which imposes a heavy treatment burden and significantly compromises patients’ quality of life. Although stem cell transplantation aims to restore pancreatic islet function, it is still in the exploratory stage with limited clinical translation [3].

The onset of T1D involves genetic susceptibility, environmental triggers, and immune dysregulation [4,5]. In recent years, an increasing number of studies have demonstrated that intestinal microbiota dysbiosis contributes critically to the development and progression of T1D [6,7]. The gut microbiota affects diabetes pathogenesis via multiple pathways, including regulating host metabolism, immune function, and intestinal barrier integrity [8,9,10]. Therefore, targeting the gut microbiota may represent a novel strategy for the prevention and treatment of T1D [11].

*Dendrobium huoshanense*, a precious traditional Chinese medicine belonging to the Orchidaceae family, possesses various pharmacological activities including hypoglycemic, anti-inflammatory, antioxidant, and immunomodulatory effects [12]. Our previous study demonstrated that *Dendrobium huoshanense* water extract (DHWE) significantly reduced fasting blood glucose, plasma C-reactive protein (CRP), and malondialdehyde (MDA) levels; increased plasma total antioxidant capacity (T-AOC); improved intestinal mucosal integrity and islet morphology; alleviated endoplasmic reticulum stress in the intestinal mucosa; and modulated the plasma metabolome in STZ-T1D model rats [13]. Our in vitro study further revealed that DHWE could beneficially reshape the gut microbiota of a STZ-T1D model rat, enhance short-chain fatty acid production, and restore a healthier microbial profile [14]. *Dendrobium huoshanense* also ameliorates gut microbiota dysbiosis in other disease models, such high-fat diet-induced obese mice [15]. However, the precise mechanisms by which *Dendrobium huoshanense* alleviates T1D via gut microbiota modulation remain insufficiently elucidated and require further investigation.

Metagenomics enables direct analysis of the entire genomic information within microbial communities without the need for isolation and cultivation of individual strains. Compared with 16S rDNA sequencing, metagenomics provides a more accurate profile of microbial composition and interactions, and allows in-depth investigation of metabolic pathways and gene functions at the molecular level [16,17]. Therefore, this study employed metagenomic sequencing to investigate the effects of DHWE on the gut microbiota of STZ-T1D rats, aiming to provide new clues regarding how gut microbial changes may contribute to the modulatory effects of *Dendrobium huoshanense* in T1D conditions.

## 2. Result

### 2.1. Analysis of Gut Microbiota Composition

#### 2.1.1. DHWE and MET Did Not Affect α-Diversity but Improved β-Diversity of Gut Microbiota

The rarefaction curve is primarily used to determine whether the microbial abundance of a sample has been sufficiently captured by sequencing. If the curves in the plot exhibit a slope approaching zero at a specific sequencing depth (x-axis), it indicates that sequencing additional reads beyond this depth is unlikely to detect new microbial features. In the present study, the rarefaction curve for α-diversity analysis showed that the curves plateaued at a sequencing depth of 1,500,000 sequences, confirming that the sequencing depth was sufficiently deep to capture the complete microbial diversity (Figure S1).

Key α-diversity indicators in metagenomic sequencing include: Shannon (reflecting richness and evenness), Simpson (probability of interspecies selection), Chao1 (estimating richness by accounting for low-abundance species), and adjusted richness estimator (ACE). Good’s coverage exceeded 99.9% across all groups (Table 1), confirming sufficient sequencing depth. α-diversity analysis revealed no significant differences in gut microbial diversity or richness between the MOD and CON groups (*p* > 0.05); neither DHWE nor MET intervention significantly altered these metrics in T1D rats (*p* > 0.05). However, the Shannon index in the MET group was significantly lower than that in the DHWE group (*p* < 0.05). Additionally, both the DHWE and MET groups exhibited significantly lower ACE values compared with the CON group (*p* < 0.05).

**Figure 1:**
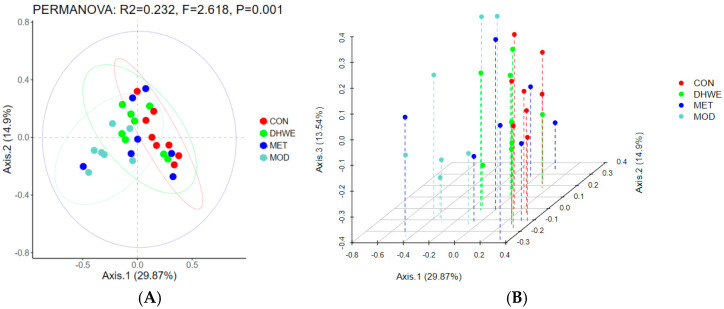
Beta-diversity of gut bacterial communities based on weighted UniFrac distance via PCoA. (A) Two-dimensional PCoA plot; (B) Three-dimensional PCoA plot. Samples with shorter spatial distance exhibit higher similarity in gut microbiota composition. PERMANOVA test confirmed significant inter-group dissimilarity (R^2^ = 0.232, F = 2.618, *p* = 0.001) among four experimental groups (CON, MOD, MET, DHWE).

PCoA is a multivariate statistical method that applies variance decomposition to reduce the dimensionality of high-dimensional microbial data, thereby extracting the most important structural patterns and variation within the data (β-diversity). As shown in Figure 1, PCoA analysis revealed a statistically significant difference in microbial community composition among the four groups (*p* = 0.001). Interventions with DHWE and MET exerted a significant effect on the gut microbiota composition of T1D rats, shifting their microbial profiles closer to that CON group relative to the MOD group. Additionally, sample points in the MET group were relatively dispersed, indicating statistically significant individual variability in gut microbial composition within this group.

#### 2.1.2. DHWE and MET Improved Gut Microbial Community Composition

In this study, metagenomic sequencing yielded a total of 145,624,491 valid clean reads. After taxonomic annotation and filtering, 1752 bacterial taxa were identified. A total of 533 bacterial taxa were shared among all groups. The MOD, DHWE, and MET groups shared an additional 105, 136, and 169 bacterial taxa with the CON group, respectively. These results indicated that DHWE and MET treatments improved the gut microbiota composition of T1D rats, rendering it closer to that of normal rats in terms of bacterial species richness (Figure S2).

**Figure 2:**
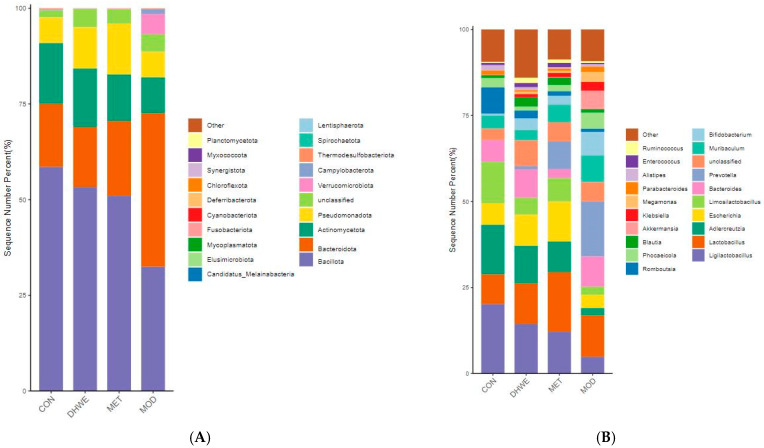

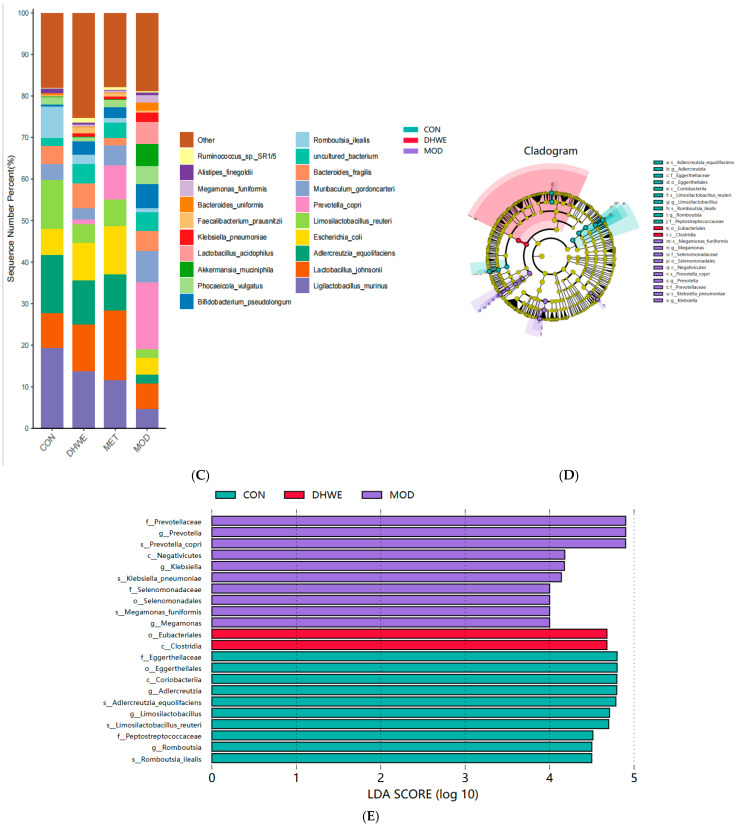
Key phylotypes of the fecal microbiota. The relative abundance of the top bacteria at different taxonomic levels across groups was visualized using stacked bar plots. (**A**) Bacterial abundance at the phylum level. (**B**) Bacterial abundance at the genus level. (**C**) Bacterial abundance at the species level. (**D**) LEfSe evolutionary tree. The cladogram corresponds to different taxonomic levels (phylum, order, family, and genus) from the inside out, with hierarchical connections representing the relationships among them. Each circular node represents a taxon; yellow indicates no significant differences among groups, while non-yellow colors indicate that the taxon is a characteristic microorganism corresponding to the colored group (with significantly higher abundance in that group). Colored fan-shaped areas indicate the subclassification intervals of characteristic microorganisms. (**E**) LEfSe bar chart. Each horizontal bar represents a taxon, and the length of the bar corresponds to the linear discriminant analysis (LDA) score. A higher LDA score indicates a greater difference. The color of the bar corresponds to the group to which the characteristic microorganism belongs, and characteristic microorganisms (biomarkers) exhibit relatively high abundance in the corresponding group. Significantly different bacteria were identified using the LEfSe method with the non-parametric Kruskal–Wallis rank sum test at a significance level of 0.05 and an LDA score (lg) > 4.

These 1752 bacterial taxa mainly consisted of 33 phyla, 55 classes, 113 orders, 225 families, and 594 genera. Overall, *Bacillota* (formerly Firmicutes, 32.4–58.5%), *Bacteroidota* (15.7–40.1%), *Actinomycetota* (9.3–15.8%), and *Pseudomonadota* (formerly Proteobacteria, 6.7–13.3%) were the dominant phyla (Figure 2A). At the genus level, the dominant genera included *Ligilactobacillus* (4.9–20.2%), *Lactobacillus* (8.7–17.3%), *Adlercreutzia* (2.1–14.4%), *Escherichia* (3.9–11.6%), *Limosilactobacillus* (2.4–12.1%), *Bacteroides* (2.5–8.8%), and *Prevotella* (0.05–16.0%) (Figure 2B). At the species level, the dominant bacterial species were *Ligilactobacillus murinus* (4.6–19.3%), *Lactobacillus johnsonii* (6.2–16.8%), *Adlercreutzia equolifaciens* (2.1–14.0%), *Escherichia coli* (3.9–11.6%), *Limosilactobacillus reuteri* (2.2–11.8%), *Prevotella copri* (0.01–15.9%), *Muribaculum gordoncarteri* (2.7–7.7%), and *Bacteroides fragilis* (1.6–6.0%) (Figure 2C). Linear discriminant analysis effect size (LEfse) was used to identify group-specific characteristic bacteria. LEfSe analysis with an LDA score > 2 showed that the CON, DHWE, MET, and MOD groups had 41, 21, 2 and 10 characteristic bacteria (with relative abundance more than twice that of other groups), respectively (Figure S3). LEfSe analysis with an LDA score > 4 (Figure 2D,E) revealed that the CON, DHWE, and MOD groups had 10, 2, and 10 characteristic bacteria (with relative abundance more than four times that of other groups), respectively. The characteristic bacteria in the CON group were *f_Eggerthellacdeae*, *o_Eggerthellales*, *c_Coriobateriia*, *g_Adlercreutzia*, *s_Adlercreutzia_equolifaciens*, *g_Limosilactobacillus*, *s_Limosilactobacillus_reuteri*, *f_Peptostreptococcaceae*, *g_Romboutsia*, and *s_Romboutsia_ilealis*; those in the DHWE group were *o_Eubacteriales* and *c_Clostridia*; and those in the MOD group were *f_Prevotellaceae*, *g_Prevotella*, *s_Prevotella_copri*, *c_Negativicutes*, *g_Klebsiella*, *s_Klebsiella_pneumoniae*, *f_Selenomonadaceae*, *o_Selenomonadales*, *s_Menamonas_funiformis*, and *g_Megamonas*. No characteristic bacteria (with relative abundance more than four times that of other groups) were observed in the MET group, indicating that MET exerts no distinct group-specific effect on the gut microbiota of T1D rats.

We further compared the differences in the relative abundance of the top 20 dominant bacterial communities among groups at the phylum, genus, and species levels, respectively. As shown in Table 2, at the phylum level, no significant overall differences in the relative abundances of *Actinomycetota*, *Bacteroidota*, and *Bacillota* were detected among the four groups (Kruskal–Wallis, all *p* > 0.05). Post hoc Dunn’s test revealed that the MOD group showed significant alterations compared with the CON group in all three phyla: *Actinomycetota* (*p* = 0.0370), *Bacteroidota* (*p* = 0.0342), and *Bacillota* (*p* = 0.0067). Additionally, the MOD group exhibited significantly higher relative abundance of *Bacteroidota* compared with the DHWE (*p* = 0.0220) and MET (*p* = 0.0195) groups. Furthermore, the ratio of *Bacteroidota* to *Bacillota* differed significantly across the four groups (Kruskal–Wallis, *p* = 0.0110), with significant pairwise differences observed between CON and MOD (*p* = 0.0122), CON and MET (*p* = 0.0078), and MOD and DHWE (*p* = 0.0151), as well as DHWE and MET (*p* = 0.0052).

At the genus level, the relative abundance of Bifidobacterium showed no significant overall difference across the four groups (Kruskal–Wallis, *p* = 0.1362). Although pairwise Dunn’s test revealed marginal differences between CON vs. MOD (*p* = 0.0239) and MET vs. MOD (*p* = 0.0181), as well as a marginal trend between DHWE vs. MOD (*p* = 0.0553) for Bifidobacterium, these pairwise differences were not statistically significant given the non-significant overall group effect. Significant intergroup variations were detected in the relative abundances of *Adlercreutzia* (*p* = 0.0023), *Limosilactobacillus* (*p* = 0.0053), *Ruminococcus* (*p* = 0.0246), *Romboutsia* (*p* = 0.0015), *Megamonas* (*p* = 0.0040), *Prevotella* (*p* = 0.0027), and *Klebsiella* (*p* = 0.0180). Post hoc Dunn’s tests indicated that, compared with the CON group, the MOD group exhibited markedly decreased abundances of *Adlercreutzia*, *Limosilactobacillus* and *Romboutsia*, whereas the abundances of *Megamonas*, Prevotella and Klebsiella were significantly increased. Compared with the MOD group, the DHWE group showed a significantly higher level of *Adlercreutzia* (*p* = 0.0030) and a lower level of *Megamonas*, while the MET group had significantly increased abundances of *Limosilactobacillus* and decreased abundance of *Megamonas* and *Prevotella*. Meanwhile, both DHWE and MET groups displayed significantly reduced Romboutsia abundance and increased Klebsiella abundance compared with the CON group.

At the species level, no significant overall differences across the four groups were found in the relative abundances of *Bifidobacterium pseudolongum* (*p* = 0.2338), *Lactobacillus johnsonii* (*p* = 0.2900), *Faecalibacterium prausnitzii* (*p* = 0.0696), and *Bacteroides uniformis* (*p* = 0.3091), with several marginal or nominal pairwise differences observed between certain groups that were not statistically significant given the non-significant overall group effect. Significant intergroup variations were detected in the relative abundances of *Adlercreutzia equolifaciens* (*p* = 0.0024), *Limosilactobacillus reuteri* (*p* = 0.0053), *Ruminococcus* sp. SR1/5 (*p* = 0.0294), *Romboutsia ilealis* (*p* = 0.0015), *Megamonas funiformis* (*p* = 0.0035), *Prevotella copri* (*p* = 0.0131), and *Klebsiella pneumoniae* (*p* = 0.0217). Post hoc Dunn’s tests indicated that, compared with the CON group, the MOD group showed significantly decreased abundances of *Adlercreutzia equolifaciens*, *Limosilactobacillus reuteri* and *Romboutsia ilealis*, while the abundances of *Megamonas funiformis*, *Prevotella copri* and *Klebsiella pneumoniae* were significantly increased. Compared with the MOD group, the DHWE group exhibited significantly restored levels of *Adlercreutzia equolifaciens* and *Megamonas funiformis*, and the MET group displayed significantly increased abundances of *Limosilactobacillus reuteri* and significantly reduced levels of *Megamonas funiformis* and *Prevotella copri*. Moreover, both the DHWE and MET groups had significantly lower abundance of *Romboutsia ilealis*, and both DHWE and MET groups showed increased levels of Klebsiella pneumoniae compared with the CON group.

The specific differences in the microbial communities of each group can be visually observed through hierarchical clustering heatmaps. From the genus-level (Figure S4A) and species-level (Figure S4B) bacterial heatmaps, it is evident that the microbial community of the MOD group is distinctly different from that of the CON group, whereas the communities of the DHWE and MET groups are more closely similar to that of the CON group than is the MOD group.

#### 2.1.3. Inter-Bacterial Correlation Analysis

**Figure 3:**
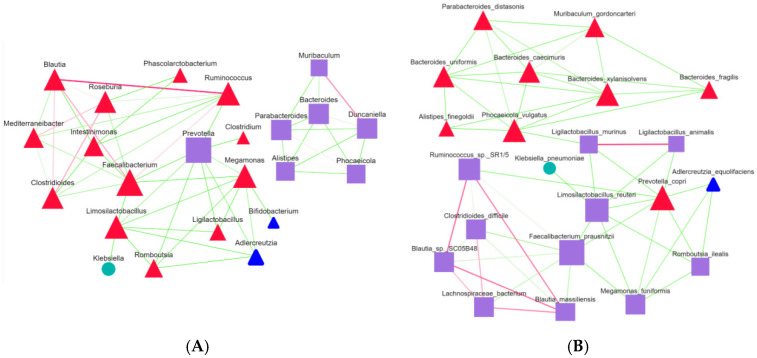
Microbial modular co-occurrence network. Different shapes (triangles, squares, circles) and colors (red, purple, blue, green) denote distinct bacterial taxonomic clusters, classified based on taxonomic affiliations. Node size corresponds to the average relative abundance of the bacterial taxa in the samples (larger nodes indicate higher abundance). Red edges indicate a positive correlation, reflecting consistent trends in the relative abundance of the two taxa; green edges indicate a negative correlation, reflecting opposing trends in relative abundance. Edge thickness corresponds to the strength and statistical significance of the correlation (thicker edges indicate stronger, more statistically significant correlations). (**A**) Genus level; (**B**) species level.

Spearman’s correlation analysis was performed on the top 30 bacterial genera or species (ranked by relative abundance) to identify interspecies interactions. Spearman’s correlation coefficients at the genus and species levels are presented in Tables S5 and S6, respectively. To visually characterize these inter-bacterial correlations, a modular co-occurrence network was constructed. At the genus level (Figure 3A), most bacterial genera exhibited significant correlations with other taxa. The top 10 genera by relative abundance were *Ligilactobacillus*, *Lactobacillus*, *Adlercreutzia*, *Escherichia, Limosilactobacillus*, *Bacteroides*, *Prevotella*, *Muribaculum*, *Bifidobacterium*, and *Romboutsia*. Among these, *Bifidobacterium* was only positively correlated with *Megamonas*, and *Klebsiella* was positively correlated with *Limosilactobacillus*; all other genera showed both positive and negative correlations with multiple taxa, indicating complex interspecies interactions within the gut microbiota. Notably, *Ruminococcus*, *Blautia*, *Mediteraneibacter*, *Roseburia*, *Intestinimonas*, *Prevotella*, *Megamonas*, *Limosilactobacillus*, *Faecalibacterium*, and *Clostridioides* were correlated with at least five other bacterial genera, suggesting they may play key roles in maintaining gut ecosystem homeostasis. Furthermore, *Muribaculum*, *Bacteroides*, *Parabacteroides*, *Duncaniella*, *Alistipes*, and *Phocaeicola* clustered within the same module, implying their potential synergistic involvement in specific metabolic or ecological processes. At the species level (Figure 3B), bacterial species were primarily clustered into two distinct modules. The first module included *Parabacteroide distasonis*, *Muribaculum gordoncarteri*, *Bacteroides uniformis*, *Bacteroides xylanisolvens*, *Bacteroides fragilis*, *Alistipes finegoldii*, and *Phocaeicola vulgatus*; the second module comprised *Ruminococcus* sp. SR1/5, *Clostridioides difficile*, *Blautia* sp. SC05B48, *Lachnospiraceae bacterium*, *Blautia massiliensis*, *Faecalibacterium prausnitzii*, *Limosilactobacillus reuteri*, *Ligilactobacillus murinus*, *Ligilactobacillus animalis*, *Megamonas funiformis*, and *Romboutsia ilealis*. Additionally, *Parabacteroides distasonis*, *Muribaculum gordoncarteri*, *Bacteroides uniformis*, *Bacteroides caecimuris*, *Bacteroides xylanisolvens*, *Phocaeicola vulgatus*, *Ruminococcus* sp. SR1/5, *Limosilactobacillus reuteri*, *Prevotella copri*, *Clostridioides difficile*, *Blautia* sp. SC05B48, *Blautia massiliensis*, *Faecalibacterium praushitzii*, and *Megamonas funiformis* were correlated with at least five other bacterial species, indicating these taxa may be critical for maintaining gut ecosystem stability.

### 2.2. Gene Function Analysis

#### 2.2.1. Differential Gene Analysis

**Figure 4:**
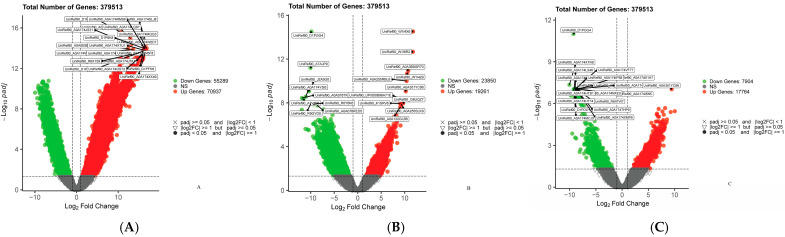
Volcano plots of differential gene expression analysis. Differential gene expression analysis was conducted with the DESeq2 method on the Wekemo Bioincloud platform. Raw gene counts based on UniRef90 protein abundance tables were normalized, and differential expression between experimental groups was tested. Results were visualized as volcano plots, with genes plotted by log_2_(fold change) (x-axis) and −log_10_(adjusted *p*-value, padj) (y-axis). Genes with |log_2_FC| ≥ 1 and padj < 0.05 were defined as significantly upregulated (red) or downregulated (green). Genes with padj ≥ 0.05 & |log_2_FC| < 1 are drawn with cross marks inside plotting area; genes with |log_2_FC| ≥ 1 & padj ≥ 0.05 are marked as hollow inverted triangles. (**A**) MOD vs. CON; (**B**) DHWE vs. MOD; (**C**) MET vs. MOD.

Differential gene analysis was conducted with the DESeq2 algorithm on the Wekemo Bioincloud platform [18]. Compared with the CON group, the MOD group had 70,937 significantly upregulated and 55,289 downregulated genes (Figure 4A). In contrast, the DHWE group had 19,261 significantly upregulated and 23,850 significantly downregulated genes relative to the MOD group (Figure 4B). Meanwhile, the MET group had 17,764 significantly upregulated and 7904 significantly downregulated genes compared to the MOD group (Figure 4C). Collectively, these results indicated that the gut microbial gene composition profiles of T1D rat models were significantly altered relative to normal rats (CON group). Furthermore, interventions with DHWE and MET markedly reversed the alterations in gut microbial gene composition profiles of T1D rats, suggesting a regulatory effect of these interventions on microbial gene function.

#### 2.2.2. KEGG Pathway Abundance

This study identified a series of KEGG pathways related to gut microbiota metabolism via metagenomic functional annotation, as well as those specific to some hosts or eukaryotes (such as small cell lung cancer and viral myocarditis, etc.). However, these host- or eukaryote-specific pathways are not inherent metabolic functions of the gut microbiota, and their emergence is mainly due to the limitations of metagenomic functional prediction algorithms: on the one hand, the KEGG database contains gene information for the entire species, and homology matching of short sequences can easily lead to incorrect mapping of host gene fragments to bacterial pathways; on the other hand, residual host epithelial cell DNA in the sample may also cause annotation bias. To ensure the reliability of the analysis results, we only retained pathways that clearly belong to bacterial metabolism (such as metabolic pathways, phenylalanine/tyrosine/tryptophan biosynthesis, etc.) in the subsequent core analysis, and provided the complete original annotation results as Supplementary Materials. This approach ensures result rigor and provides a reference for interpreting potential biases in metagenomic functional prediction.

KEGG level 1, the top-level classification in the KEGG metabolic pathway annotation system, categorizes all functional pathways into six macroscopic categories: metabolism, genetic information processing, environmental information processing, cellular processes, biological systems, and human diseases, and is used to generally describe the overall functions of the involved genes. In KEGG level 1 (Table S1), only the metabolism pathway was significantly upregulated in the MOD group as compared with the CON, DHWE, and MET groups (KW_*p*-value < 0.05, Dunn test *p*-value < 0.05), indicating that the metabolism of T1D model rats changed greatly and that DHWE or MET can regulate it toward normality. Further analysis of KEGG level 2 revealed that amino acids and glycan metabolism, as well as transport and catabolism, were significantly altered in the MOD group as compared with the CON, DHWE, and MET groups, indicating that DHWE and MET are beneficial for improving the metabolism of T1D rats. Subsequently, analysis of KEGG level 3 showed that phenylalanine, tyrosine and tryptophan biosynthesis was significantly upregulated in the MOD group as compared with the CON, DHWE, and MET groups (*p* < 0.05, KW test followed by Dunn test). Cysteine and methionine metabolism was significantly upregulated in the MOD group compared with the CON and MET groups, but no significant difference was observed between the MOD and DHWE groups (*p* < 0.05, KW test followed by Dunn test). Arginine biosynthesis was upregulated in the MOD group as compared with the MET group (*p* < 0.05, KW test followed by Dunn test). Collectively, rats in the MOD group may enhance amino acid metabolism to cope with metabolic disturbances. In addition, other glycan degradation, vitamin B_6_ metabolism, novobiocin biosynthesis, and tetracycline biosynthesis were significantly upregulated in the MOD group as compared with the CON, DHWE, and MET groups (*p* < 0.05, KW test followed by Dunn test).

**Figure 5:**
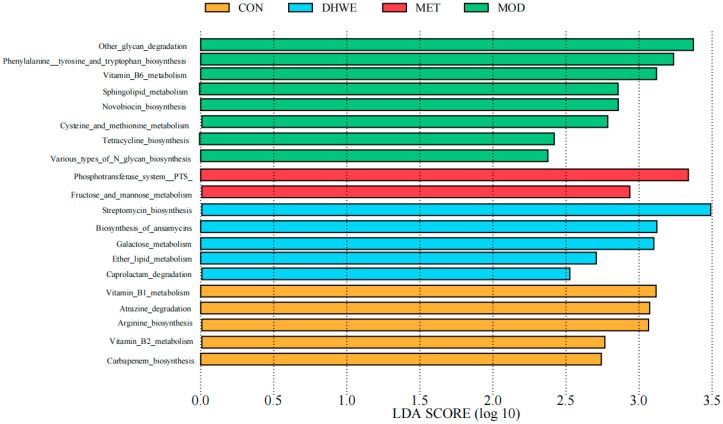
KEGG level 3 pathway analysis by LEfSe (LDA score > 2). Each bar represents a specific KEGG level 3 metabolic pathway. The higher the LDA score, the more statistically significant the enrichment of this pathway in the corresponding group (CON, DHWE, MET, or MOD).

KEGG level 3 pathway linear discriminant analysis effect size (LEfSe, LDA score > 2) (Figure 5) showed that the enriched pathways in the MOD group included other glycan degradation, phenylalanine, tyrosine and tryptophan biosynthesis, vitamin B_6_ metabolism, novobiocin biosynthesis, tetracycline biosynthesis, cysteine and methionine metabolism, various types of N-glycan biosynthesis, and sphingolipid metabolism. Among them, other glycan degradation exhibited the highest abundance in the MOD group, suggesting the significantly enhanced ability of the gut microbiota to degrade complex glycans such as mucins and dietary heteroglycans. Enrichment of aromatic amino acid synthesis (phenylalanine, tyrosine, tryptophan) and vitamin B_6_ metabolic pathways in the MOD group indicates enhanced microbial amino acid and vitamin synthesis functions, which may participate in host neurotransmitter production and oxidative stress regulation. The enrichment of antibiotic biosynthetic pathways (neomycin, tetracycline) in the MOD group suggests the proliferation of rare antibiotic-producing strains in the intestinal tract. This intensification of interspecific competition within the microbial community may further exacerbate microbial dysbiosis. Only two metabolic pathways were enriched in the MET group: the phosphotransferase system (PTS) and fructose and mannose metabolism. As the core pathway for bacterial carbohydrate transport and phosphorylation, PTS enrichment indicates a significant enhancement in the capacity of the MET group microbiota to uptake and utilize monosaccharides (e.g., glucose and fructose). The concurrent enrichment of fructose and mannose metabolism further suggests a microbial preference for dietary carbohydrate metabolism, which may correlate with improved host glucose homeostasis following MET intervention. In the DHWE group, the enriched pathways included streptomycin biosynthesis, ansamycin biosynthesis, galactose metabolism, ether lipid metabolism, and caprolactam degradation. The enrichment of antibiotic biosynthetic pathways (streptomycin) implies the proliferation of specific antibiotic-producing strains after DHWE intervention, which may modulate the microbiota structure and strengthen microbial competitive balance through antibiotic secretion. Additionally, the enrichment of galactose and ether lipid metabolism reflects enhanced metabolic capabilities toward specific carbohydrates and lipids. These pathways may participate in host metabolic regulation and intestinal barrier protection via metabolites such as short-chain fatty acids and ether lipids. The CON group was characterized by enriched pathways of vitamin B_1_ and B_2_ metabolism, atrazine degradation, arginine biosynthesis, and carbapenem biosynthesis. The enrichment of vitamin B_1_ and B_2_ metabolism indicates a robust capacity for synthesizing water-soluble vitamins, which can provide the host with abundant endogenous vitamins and reflects the core nutritional metabolic function of a healthy gut microbiota. The enrichment of the atrazine degradation pathway suggests a stronger metabolic capacity for exogenous environmental pollutants, a reflection of the metabolic plasticity inherent to a healthy microbiota. Furthermore, the enrichment of the carbapenem biosynthetic pathway indicates the presence of rare strains producing broad-spectrum antibiotics in the CON group, which contributes to maintaining the competitive balance and overall stability of the microbial community.

#### 2.2.3. Gene Enrichment Analysis

Four enrichment analysis approaches were employed to identify functionally enriched gene sets in each group: KEGG over-representation analysis (KEGG ORA), KEGG gene set enrichment analysis (KEGG GSEA), Gene Ontology over-representation analysis (GO ORA), and Gene Ontology gene set enrichment analysis (GO GSEA). KEGG ORA and GO ORA use a hypergeometric test to evaluate whether the proportion of differentially expressed genes (DEGs) mapping to a specific KEGG pathway or GO term is significantly higher than the corresponding proportion in the background gene set. A significant result indicates that the pathway or GO term is over-represented in the analyzed gene list. In contrast, KEGG GSEA and GO GSEA do not rely on a predefined DEG threshold. Instead, they detect coordinated, subtle changes in gene abundance across an entire gene set, which may collectively contribute to the functional enrichment of a specific KEGG pathway or GO term.

**Figure 6:**
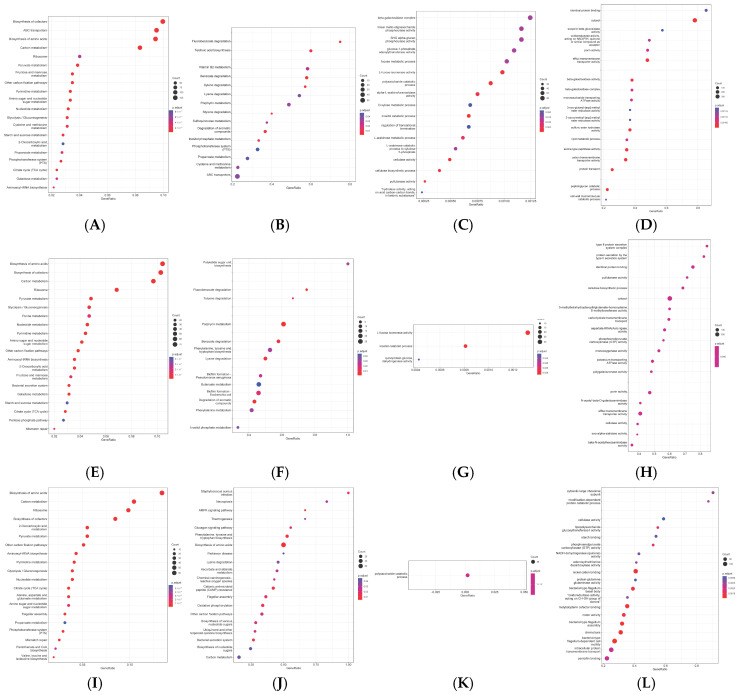
Gene enrichment analysis. For KEGG ORA and KEGG GSEA plots, the vertical axis shows pathway names sorted by enrichment significance (from top to bottom, significance decreases). For GO ORA and GO GSEA plots, the vertical axis shows GO term names. The horizontal axis represents GeneRatio (number of differentially expressed genes enriched in a pathway/total number of genes annotated in the pathway), ranging from 0.02 to 0.10. Bubble size indicates count (number of enriched differentially expressed genes), ranging from 50 to 125. Bubble color represents *p*.adjust (adjusted *p*-value), gradually changing from red (significant, *p*.adjust ≈ 2 × 10^−13^) to blue (insignificant). Genes were ranked by fold change, and enrichment significance was determined using a false discovery rate (FDR) < 0.05. Some non-bacterial KEGG or GO terms were excluded. (**A**) KEGG ORA between MOD and CON. (**B**) KEGG GSEA between MOD and CON. (**C**) GO ORA between MOD and CON. (**D**) GO GSEA between MOD and CON. (**E**) KEGG ORA between DHWE and MOD. (**F**) KEGG GSEA between DHWE and MOD. (**G**) GO ORA between DHWE and MOD. (**H**) GO GSEA between DHWE and MOD. (**I**) KEGG ORA between MET and MOD. (**J**) KEGG GSEA between MET and MOD. (**K**) GO ORA between MET and MOD. (**L**) GO GSEA between MET and MOD.

KEGG ORA of MOD vs. CON revealed that differentially abundant genes were significantly enriched in key metabolic pathways, including cofactor biosynthesis, ATP-binding cassette (ABC) transporters, and amino acid biosynthesis (Figure 6A). KEGG GSEA identified enriched genes primarily involved in bacterial xenobiotic degradation (e.g., fluorobenzoic acid and benzoate degradation) and fundamental metabolic/physiological processes (e.g., teichoic acid biosynthesis and vitamin B_2_ metabolism), reflecting the high activity of the microbial community in pollutant metabolism and self-physiological regulation (Figure 6B). GO ORA analysis highlighted carbohydrate degradation-related enzyme activities and metabolic processes as the most prominent functional characteristics. Additionally, functions with high gene proportions (e.g., β-galactosidase complexes) further indicated the core metabolic direction of the microbiota (Figure 6C). Conversely, GO GSEA showed that the cytosol was the most significantly enriched term with a high gene proportion, reflecting robust core intracellular metabolic activity within the gut microbiota (Figure 6D).

Comparisons between the DHWE and MOD groups using KEGG ORA (Figure 6E) and GSEA (Figure 6F) demonstrated significant enrichment of core primary metabolic pathways (e.g., carbohydrate and amino acid metabolism) and enhanced activity of xenobiotic degradation pathways. These results indicate improved nutrient utilization and environmental adaptability of the microbiota following DHWE intervention. GO ORA (Figure 6G) and GSEA (Figure 6H) results further revealed that functional differences between the two groups were concentrated in polysaccharide-degrading enzyme activity, membrane transporter activity, and molecular functions/biological processes related to intracellular metabolism. Notably, polysaccharide degradation and nutrient transport functions were more prominent in the DHWE group. Collectively, these findings demonstrate that DHWE intervention significantly remodels the metabolic functional spectrum of the gut microbiota. By enhancing the microbiota’s capacity to degrade and utilize complex carbohydrates, DHWE optimizes microbe–host nutritional interactions and improves the metabolic homeostasis of the gut microbiota.

KEGG ORA (Figure 6I) and GSEA (Figure 6J) results showed that, compared with the MOD group, the MET group exhibited significant enrichment of core primary metabolic pathways, such as amino acid metabolism and carbohydrate metabolism, in the gut microbiota. Additionally, pathways related to energy metabolism and redox homeostasis were more active, indicating that metformin intervention can effectively restore the fundamental metabolic functions of the microbiota in the T1D model. GO ORA (Figure 6K) and GSEA (Figure 6L) results revealed that functional differences between the two groups were concentrated in molecular functions and biological processes associated with enzyme activity regulation, protein transport, and intracellular metabolism. Notably, the MET group showed significantly enhanced functions such as oxidoreductase activity and protein transmembrane transport, suggesting that the microbiota adapts to the post-intervention intestinal microenvironment by optimizing metabolic enzyme activity and protein sorting capabilities. Collectively, these findings demonstrate that metformin improves nutrient utilization efficiency and redox homeostasis regulation by remodeling the metabolic functional spectrum of the gut microbiota. This, in turn, alleviates the intestinal microecological metabolic disorder in the T1D model, providing functional support for the microbiota-mediated hypoglycemic mechanism of metformin.

### 2.3. Correlations of Gut Microbiota with Serum Biochemical Indicators

**Figure 7:**
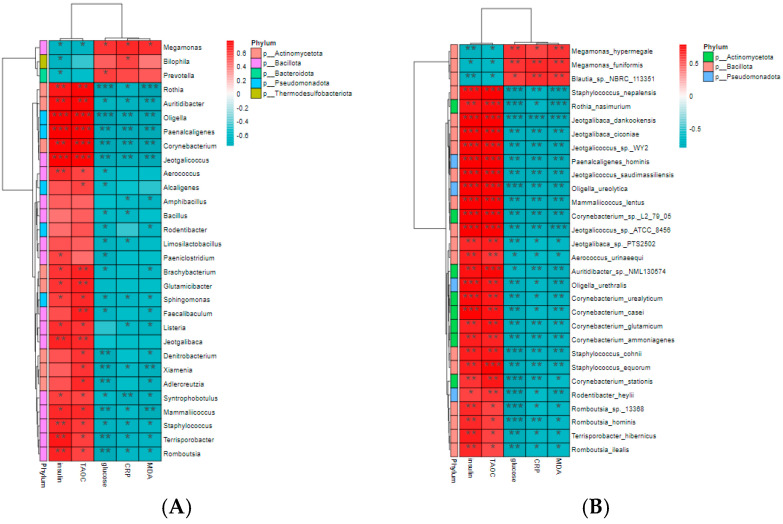
Correlation analysis between gut microbiota and blood indices. (**A**) At the genus level. (**B**) At the species level. Red and blue colors represent positive and negative correlations, respectively. Asterisks indicate statistical significance (FDR-adjusted *p* * < 0.05, FDR-adjusted *p* ** < 0.01, FDR-adjusted *p* *** < 0.001).

To further explore the relationship between gut microbiota and T1D-related phenotypes, correlation analyses were performed between gut microbiota and blood glucose, plasma CRP, MDA, and T-AOC. The results (Figure 7A) showed that the genus *Megamonas* was negatively correlated with T-AOC and plasma insulin level (FDR-adjusted *p* < 0.05), and positively correlated with blood glucose, plasma CRP, and MDA levels (FDR-adjusted *p* < 0.05). *Bilophila* was positively correlated with plasma CRP level (FDR-adjusted *p* < 0.05) and negatively correlated with plasma insulin level (FDR-adjusted *p* < 0.05). *Prevotella* was positively correlated with blood glucose level (FDR-adjusted *p* < 0.05) and negatively correlated with plasma insulin level (FDR-adjusted *p* < 0.05). *Rothia*, *Auritidibacter*, *Oligella*, *Paenalcaligenes*, *Corynebacterium*, *Jeotgalicoccus*, *Brachybacterium*, *Sphingomonas*, *Syntrophobotulus*, *Mammalicoccus*, *Staphylococcus*, *Terrisporobacter*, and *Romboutsia* were positively correlated with T-AOC and plasma insulin level (FDR-adjusted *p* < 0.05, 0.01, or 0.001), and negatively correlated with blood glucose, CRP, and MDA levels (FDR-adjusted *p* < 0.05, 0.01, or 0.001). Other genera, including *Aerococcus*, *Alcaligenes*, *Amphibacillus*, *Bacillus*, *Rodenticibacter*, *Limosilactobacillus*, *Paeniclostridium*, *Glutamicibacter*, *Faecalibaculum*, *Listeria*, *Jeotgalibacillus*, *Denitrobacterium*, *Xiamenia*, and *Adlercreutzia*, showed significant positive or negative correlations with plasma insulin, T-AOC, blood glucose, CRP, or MDA levels (FDR-adjusted *p* < 0.05 or 0.01).

Results of the correlation analysis between gut microbiota at the species level and blood indices (Figure 7B) showed that *Megamonas hypermegale*, *Megamonas funiformis*, and *Blautia* sp. NBRC 113351were negatively correlated with T-AOC and plasma insulin level (FDR-adjusted *p* < 0.05 or 0.01), and positively correlated with blood glucose, plasma CRP, and MDA levels (FDR-adjusted *p* < 0.05 or 0.01). In total, 27 bacterial species were positively correlated with T-AOC and plasma insulin level (FDR-adjusted *p* < 0.05, 0.01, or 0.001), and negatively correlated with blood glucose, plasma CRP, and MDA levels (FDR-adjusted *p* < 0.05, 0.01, or 0.001). Most of these species were positively correlated with T1D-improving indicators (plasma insulin and T-AOC) and negatively correlated with T1D-aggravating indicators (blood glucose, MDA, and CRP). Although the underlying mechanism remains unclear, it may reflect a self-regulatory compensatory response of the intestinal flora to hyperglycemia in the early stage of T1D.

### 2.4. Joint Analysis Results of Gut Microbiota Metagenome and Plasma Metabolome

**Figure 8:**
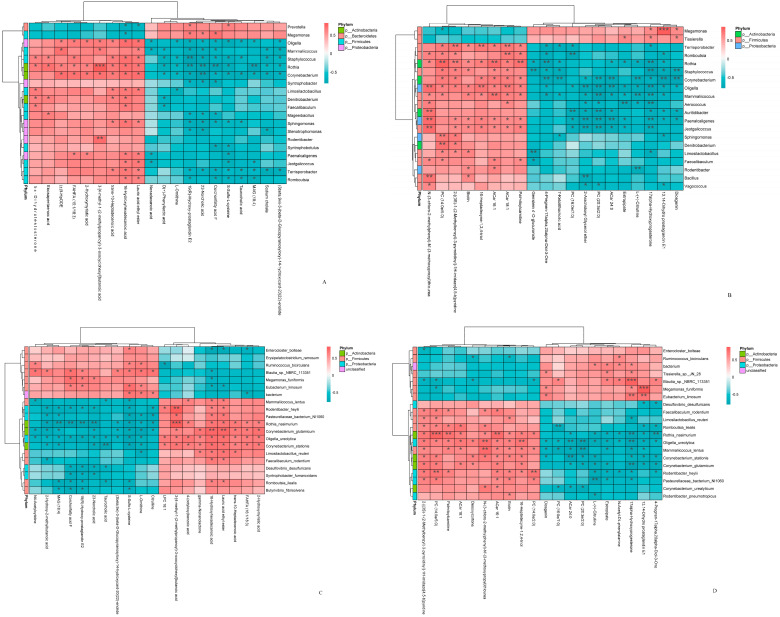
Correlation heatmap between bacterial relative abundance and metabolites detected by metabolome. (**A**) bacteria at genus level and metabolites at negative ion pattern metabolome. (**B**) bacteria at genus level and metabolites at positive ion pattern metabolome. (**C**) bacteria at species level and metabolites at negative ion pattern metabolome. (**D**) bacteria at species level and metabolites at positive ion pattern metabolome. Spearman’s correlation coefficients were calculated, and significance was assessed by permutation testing. The resulting heatmap visualized the correlation patterns with color gradients indicating positive (red) or negative (blue) associations. FDR-adjusted *p* * < 0.05, FDR-adjusted *p* ** < 0.01,FDR-adjusted *p* *** < 0.001.

We performed univariate correlation analysis between microbial features (at the genus level) and plasma metabolites using the ‘Correlation heatmap’ module in Wekemo Bioincloud [18]. The correlation heatmaps (Figure 8A,B) revealed significant associations between gut microbiota at the genus level and host plasma metabolites. Notably, *Prevotella* showed positive correlations with S-sulfo-L-cysteine and 19(R)-hydroxy-prostaglandin E_2_ (*r* > 0.5, FDR-adjusted *p* < 0.05), and negative correlations with lauric acid ethyl ester and 16-hydroxyhexadecanoic acid (r < −0.5, FDR-adjusted *p* < 0.05). *Megamonas* was significantly positively correlated with corchorifatty acid F, 23-norcholic acid, 19(R)-hydroxy-prostaglandin E_2_, 17α-hydroxyprogesterone, 13,14-dihydro-prostaglandin E_1_, and diosgenin (*r* > 0.5, FDR-adjusted *p* < 0.05), and negatively correlated with 16-hydroxyhexadecanoic acid and PC (14:0e/5:0) (*r* < −0.5, FDR-adjusted *p* < 0.05). *Tissierella* was significantly positively correlated with diosgenin, 17α-hydroxyprogesterone, and estropipate (*r* > 0.5, FDR-adjusted *p* < 0.05). Other genera, including *Terrisporobacter*, *Romboutsia*, *Rothia*, *Staphylococcus*, *Corynebacterium*, *Oligella*, *Mammaliicoccus*, *Aerococcus*, *Auritidibacter*, *Paenalcaligenes*, *Jeotgalicoccus*, *Sphingomonas*, *Denitrobacterium*, *Limosilactobacillus*, *Faecalibaculum*, *Rodentibacter*, *Bacillus*, *Vagococcus*, *Syntrophobacter*, *Mageeibacillus*, *Stenotrophomonas*, and *Syntrophobotulus*, exhibited a completely opposite microbial–metabolite correlation pattern to that of *Prevotella*, *Megamonas*, and *Tissierella*. Notably, *Oligella, Mammaliicoccus*, *Staphylococcus*, *Rothia*, *Corynebacterium*, *Limosilactobacillus*, *Denitrobacterium*, *Sphingomonas*, *Paenalcaligenes*, *Jeotgalicoccus*, *Terrisporobacter*, *Romboutsia*, *Faecalibaculum*, *Auritidibacter*, *Aerococcus*, and *Bacillus* were correlated with at least five or more plasma metabolites, indicating that they may exert complex influences on host metabolism.

To investigate the relationship between specific bacterial species and plasma metabolites, we further analyzed the correlation heatmap between microbial features (at the species level) and metabolites. The results (Figure 8C,D) showed that *Enterocloster bolteae*, *Ruminococcus bicirculans*, *Tissierella* sp. *JN 28*, *Blautia* sp. NBRC 113351, *Megamonas funiformis*, and *Eubacterium limosum* were negatively correlated with several metabolites, including fatty acid esters of hydroxy fatty acids (FAHFA) (16:1/18:3), lauric acid ethyl ester, 16-hydroxyhexadecanoic acid, lysophosphatidylcholine (LPC) 16:1, phosphatidylcholine (PC) (14:0e/2:0), biotin, desoxycortone, PC (14:0e/5:0), and 2-[(3S)-1-(2-methylbenzyl)-3-pyrrolidinyl]-1H-imidazo [4,5-b] pyridine. Conversely, these species were positively correlated with metabolites such as citrulline, L-ornithine, S-sulfo-L-cysteine, (3β,9α)-3-(β-D-glucopyranosyloxy)-14-hydroxycard-20(22)-enolide, 23-norcholic acid, 19(R)-hydroxy-prostaglandin E_2_, corchorifatty acid F, 2-hydroxy-2-methylbutanoic acid, diosgenin, N4-acetylcytidine, PC (16:0e/7:0), L-(+)-citrulline, estropipate, 13,14-dihydro-prostaglandin E_1_, 17α-hydroxyprogesterone, and N-acetyl-DL-phenylalanine. In contrast, *Desulfovibrio desulfuricans*, *Faecalibaculum rodentium*, *Limosilactobacillus reuteri*, *Romboutsia ilealis*, *Rothia nasimurium*, *Oligella ureolytica*, *Mammaliicoccus lentus*, *Corynebacterium stationis*, *Corynebacterium glutamicum*, *Corynebacterium urealyticum*, *Rodentibacter heylii*, *Rodentibacter pneumotropicus*, *Pasteurellaceae bacterium NI1060*, *Syntrophobacter fumaroxidans*, and *Butyrivibrio fibrisolvens* were positively correlated with metabolites including 2-hydroxymyristic acid, FAHFA(16:1/18:3), trans-10-heptadecenoic acid, lauric acid ethyl ester, 16-hydroxyhexadecanoic acid, γ-nonanolactone, 4-(octyloxy)benzoic acid, 3-[4-methyl-1-(2-methylpropanoyl)-3-oxocyclohexyl]butanoic acid, LPC 16:1, PC (14:0e/2:0), 16-heptadecyne-1,2,4-triol, biotin, Acylcarnitine (ACar) 16:1, N-(3-chloro-2-methylphenyl)-N-(3-methoxypropyl) thiourea, desoxycortone, ACar 18:1, palmitoylcarnitine, PC (14:0e/5:0), and 2-[(3S)-1-(2-methylbenzyl)-3-pyrrolidinyl]-1H-imidazo [4,5-b]pyridine. These species were negatively correlated with metabolites such as citrulline, L-ornithine, S-sulfo-L-cysteine, (3β,9ξ)-3-(β-D-glucopyranosyloxy)-14-hydroxycard-20(22)-enolide, taurocholic acid, 23-norcholic acid, 19(R)-hydroxy-prostaglandin E_2_, corchorifatty acid F, MAG(18:4), 2-hydroxy-2-methylbutanoic acid, N4-acetylcytidine, 4-pregnen-17α,20α-diol-3-one, 13,14-dihydro-prostaglandin E1, 17α-hydroxyprogesterone, N-acetyl-DL-phenylalanine, estropipate, L-(+)-citrulline, PC(20:3e/2:0), ACar 24:0, PC (16:0e/7:0), and diosgenin. Notably, all the aforementioned species, including *Blautia* sp. NBRC 113351, *Butyrivibrio fibrisolvens*, *Corynebacterium stationis*, *Corynebacterium glutamicum*, *Corynebacterium urealyticum*, *Desulfovibrio desulfuricans*, *Enterocloster bolteae*, *Eubacterium limosum*, *Faecalibaculum rodentium*, *Limosilactobacillus reuteri*, *Mammaliicoccus lentus*, *Megamonas funiformis*, *Oligella ureolytica*, *Pasteurellaceae bacterium* NI1060, *Rodentibacter heylii*, *Rodentibacter pneumotropicus*, *Romboutsia ilealis*, *Rothia nasimurium*, *Ruminococcus bicirculans*, *Syntrophobacter fumaroxidans*, and *Tissierella* sp. JN 28, were correlated with at least five or more plasma metabolites.

## 3. Discussion

The gut microbiota, an integral component of the animal body, has co-evolved with the host over millennia. Through continuous individual adaptation and natural selection, the microbiota maintains a dynamic equilibrium with the host and among its own constituent species, forming an interdependent and mutually constrained ecosystem. Under physiological conditions, the relatively stable structure and function of this ecosystem are pivotal for sustaining host health. In recent years, accumulating evidence has linked intestinal dysbiosis to a spectrum of disorders, including autoimmune diseases, obesity, diabetes, and depression [19,20,21,22]. Specifically, alterations in the gut microbiota play a crucial role in the pathogenesis of T1D. For instance, STZ fails to induce T1D in antibiotic-depleted mice but readily triggers the disease in microbiota-replete controls [23]. Mechanistically, bacterial translocation to pancreatic lymph nodes activates nucleotide-binding oligomerization domain-containing protein 2 (NOD2) receptors on myeloid cells, leading to the secretion of proinflammatory cytokines (IL-1 β, IL-6, IL-23, and IL-12). This cascade promotes the differentiation of pathogenic T helper 1 (Th1) and T helper 17 (Th17) cells while suppressing regulatory T (Treg) cell generation, thereby facilitating T1D development [23]. Against the backdrop, the present study aimed to explore the effects of DHWE on the composition and function of the gut microbiota in T1D rats, with the goal of providing metagenomic insights into gut microbiota alterations following DHWE intervention in STZ-T1D Rats. Our results demonstrate that DHWE intervention reshapes gut microbiota composition and function in T1D rats, which might contribute to the improvement of diabetic phenotypes.

Dominant bacterial species with high relative abundance may play a more critical role in maintaining gut microbiota homeostasis. Metagenomic analysis enables precise typing at the species or even strain level. Among the top 20 dominant species, the relative abundances of *Limosilactobacillus reuteri*, *Adlercreutzia equolifaciens*, and *Romboutsia ilealis* were significantly decreased, while those of *Megamonas funiformis*, *Prevotella copri* and *Klebsiella pneumoniae* were increased in the MOD group compared with the CON group (*p* < 0.01 or 0.001). Both DHWE and MET interventions tended to reverse these changes in the relative abundance of these bacteria in T1D rats.

LEfSe analysis revealed that *f_Prevotellaceae*, *g_Prevotella*, *s_Prevotella_copri*, *c_Negativicutes*, *g_Klebsiella*, *s_Klebsiella_pneumoniae*, *f_Selenomonadaceae*, *o_Selenomonadales*, *s_Megamonas_funiformis*, and *g_Megamonas* were characteristic taxa in the MOD group, with relative abundances more than four times higher than in other groups. This implies that these bacteria may be involved in the occurrence and development of T1D.

*Klebsiella pneumoniae* is a major opportunistic pathogen responsible for a broad spectrum of diseases, including urinary tract infections, cystitis, pneumonia, surgical wound infections, endocarditis, septicemia, necrotizing pneumonia, pyogenic liver abscesses and endogenous endophthalmitis [24]. In the present study, *Klebsiella_pneumoniae* was identified as both a dominant species and a characteristic taxon in the MOD group, suggesting that it may play an important role in the pathogenesis of T1D. This finding is consistent with a previous study by Liu et al. [25], who reported enrichment of *Klebsiella pneumoniae* in T1D patients. Additionally, it has been documented that type 2 diabetes can enhance the pathogenicity of *Klebsiella pneumoniae* [26]. Therefore, we hypothesize that the diabetic environment in T1D may facilitate the intestinal colonization and proliferation of *Klebsiella pneumoniae*, leading to its increased abundance. Notably, our correlation analysis revealed no significant association between *Klebsiella pneumoniae* and either plasma biochemical indicators or metabolomic profiles. Furthermore, the species-level bacterial correlation network (Figure 3B) showed that *Klebsiella pneumoniae* had almost no connections with other species, except for a negative correlation with *Limosilactobacillus_reuteri* (*r* = −0.547, *p* = 0.017). Based on these observations, we speculate that the elevated abundance of *Klebsiella pneumoniae* in the MOD group may be primarily driven by the favorable diabetic gut environment, while *Limosilactobacillus reuteri* may exert an inhibitory effect on its proliferation. Future studies should investigate the impact of pre-inoculation with *Klebsiella pneumoniae* on the success rate of T1D modeling, to determine whether this bacterium directly promotes the onset of T1D.

*Bifidobacterium_pseudolongum* is a well-known probiotic. This species has been reported to reduce triglyceride levels by modulating the gut microbiota in high-fat diet-fed mice [27], attenuate cardiac fibrosis development in mice [28], and suppress non-alcoholic fatty liver disease-associated hepatocellular carcinoma by producing acetate [29]. Our results showed that the relative abundance of *Bifidobacterium pseudolongum* tended to increase in the MOD group, which was consistent with the findings of Ma et al. [30]. Although *Bifidobacterium pseudolongum* is generally recognized as beneficial commensals at the species level in healthy individuals, their enrichment in the T1D model group and positive correlation with hyperglycemia might imply intraspecies functional heterogeneity. Even without strain-level resolution, it is reasonable to infer that distinct intraspecies variants may exert divergent roles under healthy versus diabetic pathological conditions. In addition, gut microbes might exert disease-stage-dependent bidirectional effects during T1D progression. These taxa might also act as opportunistic pathobionts in late-stage hyperglycemic rats rather than playing protective roles. Therefore, a plausible explanation is that this change may reflect a self-regulatory response of the gut microbiota during the early stage of diabetes, and may also serve as a potential marker for early-stage diabetes [30].

To our surprise, in contrast to the merely increasing trend of *Bifidobacterium pseudolongum*, the relative abundance of the potential probiotic *Megamonas funiformis* was significantly elevated in the MOD group and was identified as one of the characteristic species. *Megamonas funiformis* is an anaerobic bacterium belonging to the family *Veillonellaceae* that thrives in low-oxygen environments [31]. This species has been reported to prevent or ameliorate multiple metabolic diseases, including obesity, diabetes, atherosclerosis, cardiovascular diseases, hyperuricemia, and metabolic dysfunction-associated fatty liver disease (MAFLD) [32,33]. Yang et al. [33] demonstrated that oral administration of *Megamonas funiformis* CML154 alleviated MAFLD in high-fat diet-fed hens and mice via propionate-mediated activation of the adiponectin-adenosine 5′-monophosphate (AMP)-activated protein kinase-peroxisome proliferator-activated receptor α (APN-AMPK-PPARα) signaling pathway, thereby inhibiting de novo fatty acid synthesis and promoting β-oxidation. These findings indicated that *Megamonas* contributes to the anti-MAFLD effect of inulin. In the present study, correlation analysis with blood biochemical indices revealed that *Megamonas funiformis* was strongly negatively correlated with plasma insulin level and T-AOC, but positively correlated with plasma CRP, MDA, and blood glucose levels (Figure 7B). Integrated metagenomic and metabolomic analysis showed that *Megamonas funiformis* was positively correlated with 23-norcholic acid, 19(R)-hydroxy-prostaglandin E_2_ (19(R)-OH-PGE_2_), 13,14-dihydro-prostaglandin E_1_, and 17α-hydroxyprogesterone, while negatively correlated with 16-hydroxyhexadecanoic acid and PC (14:0e/5:0) (Figure 8C,D). 23-Norcholic acid is a bile acid that promotes tumor progression and immune evasion by regulating the farnesoid X receptor (FXR) in hepatocellular carcinoma [34]. FXR is known to modulate the production of various inflammatory cytokines [35], and early disruption of bile acid metabolism may represent one of the risk factors and pathogenic mechanisms underlying T1D [36]. 19(R)-OH-PGE_2_ is a metabolite of prostaglandin E_2_ (PGE_2_) and a highly selective agonist of the E-prostanoid receptor 2 (EP_2_) [37]. However, the precise role of PGE_2_ in autoimmune inflammation remains controversial [38]. Further research is therefore needed to clarify whether 19(R)-OH-PEG_2_ promotes or protects against T1D development. Dong et al. [39] reported higher abundances of *Prevotella copri* and *Megamonas funiformis* in women with polycystic ovary syndrome-insulin resistance (PCOS-IR) compared with PCOS-non-insulin-resistant (PCOS-NIR) individuals (LDA score > 3), and these taxa correlated with fecal propionate levels (adjusted *R*^2^ = 0.145, *p* < 0.001). Consistent with this, *Megamonas funiformis* was more abundant in individuals with pre-diabetes than in healthy or diabetic subjects [40]. These findings strongly suggest that *Megamonas funiformis* may play a specific role in the early stage of T1D. Notably, our previous in vitro fermentation study showed that DHWE increased the relative abundance of *Megamonas* in fecal microbiota from T1D rats [14]. However, in the present in vivo study, DHWE intervention significantly reduced the relative abundance of *Megamonas funiformis* compared with the MOD group (*p* < 0.01). While inter-microbial competition occurs in both experimental systems, the in vivo diabetic gut involves multiple host-specific factors that are absent in the simplified in vitro setting, such as T1D-induced intestinal inflammation, impaired intestinal barrier function, altered gut transit time, and host immune–microbe interactions. These pathological conditions may modify the survival advantage of *Megamonas funiformis* and other *Megamonas* species under DHWE intervention. In addition, our in vitro analysis was performed at the genus level, whereas the current metagenomic data resolves responses at the species level. Divergent effects of DHWE on different *Megamonas* species could further contribute to this inconsistency. Furthermore, the species-level bacterial correlation network (Figure 3B) showed that *Megamonas funiformis* was negatively associated with six genera, indicating complex interactions with other members of the microbiota. Further studies are needed to clarify whether the increased abundance of *Megamonas funiformis* in pre-diabetes or early T1D represents a driving force for disease progression, or merely a compensatory response to the disturbed intestinal environment. Targeted elimination of *Megamonas funiformis* using specific bacteriophages or other strategies, followed by assessment of STZ-T1D incidence, may help define its causal role in the early pathogenesis of T1D.

*Prevotella copri* was also identified as a dominant species with higher abundance in the MOD group compared with the CON group. Consistent with our findings, numerous studies have reported the enrichment of *Prevotella copri* in patients with T1D [41,42,43] or T2D [44]. Mechanistically, *Prevotella* species predominantly activate toll-like receptor 2 (TLR2), inducing the polarization of Th17 CD^4+^ T cells and the secretion of proinflammatory cytokines. Furthermore, *Prevotella* spp. can stimulate epithelial cells to secrete IL-8 and IL-6, thereby promoting Th17 responses and neutrophil recruitment [45]. This *Prevotella*-mediated gut mucosal inflammation increases intestinal permeability and facilitates the translocation of bacterial products, ultimately amplifying systemic inflammation [45]. In the present study, *Prevogella* at the genus level was positively correlated with plasma glucose levels and negatively correlated with plasma insulin levels (Figure 7A). However, this correlation was not observed for *Prevotella copri* at the species level. Similarly, the genus *Prevogella*—but not the species *Prevotella copri*—was positively correlated with plasma S-sulfo-L-cysteine and 19(R)-hydroxy-prostaglandin E_2_, and negatively correlated with lauric acid ethyl ester and 16-hydroxyhexadecanoic acid (Figure 8A). These results indicate that, although *Prevotella* influences T1D progression, its impact is less pronounced than that of *Megamonas*. We observed considerable inter-individual variation in the relative abundances of *Prevotella* and *Prevotella_copri*. MET interventions significantly reduced while DHWE interventions tended to reduce the abundance of *Prevotella* at both the genus and species levels (Table 1, *p* < 0.05 or 0.01), suggesting that this taxon may contribute to the severity of T1D. However, it is crucial to note the functional heterogeneity within this genus; for instance, *Prevotella histicola* has been shown to delay the onset of T1D [46]. Furthermore, our previous in vitro fermentation study demonstrated that DHWE increased the abundance of *Prevotella* in the fecal microbiota of T1D rats [14], which contrasts with its suppressive effect on *Prevotella* abundance observed in the present in vivo study. This inconsistency between in vivo and in vitro results may be similar to that of *Megamonas*. Spearman correlation analysis revealed that *Prevotella* was significantly negatively correlated with *Ligilactobacillus*, *Adlercreutzia*, *Limosilactobacillus*, *Romboutsia*, and *Clostridium*—all considered relatively beneficial taxa except *Clostridium*—and significantly positively correlated with *Megamonas*, *Ruminococcus*, and *Faecalibacterium* (Table S5). Collectively, these findings highlight the complex ecological and functional role of *Prevotella* in T1D, whose precise mechanisms require further investigation.

Although *c_Negativicutes*, *o_Selenomonadales* and *f_Selenomonadaceae* were not dominant taxa, they served as characteristic biomarkers in the MOD group, with relative abundance more than four times higher than those in other groups. To date, no robust evidence has directly linked these taxa to diabetes. A mendelian randomization study reported that higher abundances of *c_Negativicutes* and *o_Selenomonadales* were associated with an increased risk of endometriosis [47]. The precise roles of *c_Negativicutes*, *o_Selenomonadales*, and *f_Selenomonadaceae* in the initiation and progression of T1D warrant further investigation.

The relative abundance of *Adlercreutzia equolifaciens*, *Limosilactobacillus reuteri,* and *Romboutsia ilealis* were significantly lower in the MOD group than in the CON group (*p* < 0.001), which was consistent with observations at the genus level (Table 1). DHWE and MET interventions tended to increase the relative abundances of these species. *Adlercreutzia equolifaciens* [48,49], *Limosilactobacillus reuteri* [50,51], and *Romboutsia ilealis* [52] are recognized as beneficial bacteria. Notably, correlation analysis revealed that the genus *Adlercreutzia* was significantly positively correlated with plasma T-AOC and negatively correlated with blood glucose and plasma MDA levels. The genus *Limosillactobacillus* only showed a significant negative correlation with blood glucose and plasma CRP levels, but no significant positive correlation with plasma insulin or T-AOC. At the species level, neither *Adlercreutzia equolifaciens* nor *Limosillactobacillus reuteri* exhibited significant correlations with the above plasma indices. In contrast, the genus *Romboutsia* was significantly positively correlated with plasma insulin level (*p* < 0.01) and T-AOC (*p* < 0.05), and negatively correlated with plasma glucose (*p* < 0.01), CRP, and MDA level (*p* < 0.05). The correlations between the genus *Romboutsia* and plasma biochemical indices were mainly driven by three species: *Romboutsia ilealis*, *Romboutsia hominis*, and *Romboutsia* sp. 13368. Collectively, these observations suggest that *Romboutsia* may play a more important role in T1D progression than *Adlercreutzia* and *limosillactobacillus*.

Correlation analysis also revealed that some bacteria with low abundance exhibited strong correlations with plasma metabolomic and biochemical indices. Clearly, the roles of these taxa in T1D warrant further investigation. In addition, as shown in the heatmaps of correlations between bacteria and plasma indices (Figure 7A,B) or plasma metabolites (Figure 8A–D), a large proportion of identified bacteria displayed significant positive associations with indicators related to T1D amelioration, and significant negative associations with markers linked to T1D exacerbation. This may reflect a compensatory response of the gut microbiota that attempts to alleviate the metabolic disorder during T1D development.

Dynamic changes in gut microbiota composition occur during the progression of T1D. Zhu et al. [53] reported that STZ-T1D rats exhibited a significant decrease in the relative abundance of *lactobacillus* and a marked increase in *pseudomonas* after 4 weeks of modeling. At 12 weeks, the relative abundances of *Lactobacillus*, *Bacteroidetes*, and *Ruminococcus* were significantly increased, whereas that of *Bifidobacterium* was significantly decreased. Patterson et al. [54] also established an STZ-T1D rat model and observed distinct temporal shifts: one week after STZ injection, the proportions of *Parabacteroides* and *Mucispirillum* in fecal microbiota were significantly increased, while *Ruminococcus* was significantly decreased. Two weeks after STZ injection, *Ruminococcus* remained significantly reduced and *Mucispirillum* remained elevated; the abundance of *Bacteroides*, *Lactobacillus*, *Turicibacter*, and *Clostridium* was significantly increased, whereas *Parabacteroides* and members of *Ruminococcae incortae sedis* were decreased. Four weeks after STZ administration, the proportions of *Parasutterella*, *Bifidobacterium*, *Bacteroides*, and *Phascolarctobacterium* were higher, while those of *Alistipes*, *Ruminococcus*, *Ruminococcaceae incertae sedis*, *Peptostreptococcaceae incertae sedis*, and *Mucispirillum* were lower. Collectively, these studies suggest that the gut microbiota exhibits dynamic alterations in the course of T1D development. In the present study, the abundance of *Bifidobacterium* tended to increase in T1D rats on day 28 after modeling than in the CON group, which is consistent with the findings of Patterson et al. [54]. Future studies with longer longitudinal sampling are warranted to characterize gut microbiota dynamics in T1D patients, which will facilitate the development of targeted microbiota intervention strategies.

Analysis of KEGG pathway enrichment revealed that different treatments significantly altered the metabolic functional profiles of the gut microbial community. The pathway ‘other glycan degradation’ showed the highest abundance in the MOD group. This suggests that the microbiota in the MOD group may exhibit enhanced degradation of intestinal complex glycans (e.g., mucin glycans and dietary heteroglycans), thereby impairing the intestinal mucus layer and barrier function. Another notable finding was the enrichment of antibiotic biosynthetic pathways (neomycin, tetracycline) in the MOD group, which may disrupt intestinal microecological homeostasis and contribute to the reduction in beneficial bacteria. In the MET group, only two metabolic pathways were enriched: PTS and fructose and mannose metabolism. This is consistent with the absence of characteristic bacteria with more than fourfold higher abundance relative to other groups in the MET group. Our previous in vitro study showed that metformin increased the relative abundance of *Citrobacter*, *Ruminococcus*, and *Klebsiella*, while decreasing that of *Bacteroides*, *Oscillospira*, *Escherichia*, *Sutterella*, *Dorea*, *Enterocloster*, and *Proteus* [14]. Together, these findings imply that the ameliorative effect of metformin on T1D may rely mainly on its direct pharmacological action rather than an indirect effect mediated by gut microbiota modulation. In contrast, the DHWE group exhibited enrichment of numerous metabolic pathways, including streptomycin biosynthesis, ansamycins biosynthesis, galactose metabolism, ether lipid metabolism and caprolactam degradation. This indicates that DHWE exerts a more pronounced regulatory effect on the gut microbioita of STZ-T1D rats. The CON, MOD, DHWE and MET groups exhibited distinct enrichment of antibiotic biosynthesis pathways. *Ansamycins*, *carbapenem*, *novobiocin*, *streptomycin* and *tetracycline* biosynthesis pathways are not carried by dominant intestinal microbes (e.g., *Bacillota* and *Bacteroidota*), but are primarily harbored by low-abundance commensal *Streptomyces* (*Actinomycetota*) that naturally colonize the animal gut [55]. These antibiotic biosynthesis pathways might mainly function in inter-bacterial competition to suppress opportunistic pathogens and shape gut microbial communities, with negligible impacts on the host. This might represent one potential mechanism underlying gut dysbiosis in T1D rats. Further studies are required to determine whether the synthesis of different classes of antibiotics by *Streptomyces* is involved in regulating the gut microbiota structure in T1D.

## 4. Materials and Methods

### 4.1. Preparation of DHWE and Analysis of Its Components

The preparation of DHWE and analysis of its components were performed as described in our previously published paper [14]. Briefly, the stems of *Dendrobium huoshanense* were dried at 65 °C, followed by crushing and sieving through a 60-mesh sieve. Twenty grams of *Dendrobium huoshanense* powder were extracted with 800 mL of distilled water for three consecutive times. The three extracts were combined and concentrated using a rotary evaporator (FE-52AA; Shanghai Yarong Biochemical Instrument Factory, Shanghai, China) at 80 °C, and subsequently lyophilized with a vacuum freeze dryer (FD-1A-50; Beijing Boyikang Instrument Co., Ltd., Beijing, China) to obtain DHWE. The contents of carbohydrates, total polyphenols, and proteins in DHWE were determined by the phenol–sulfuric acid method, Folin–Ciocalteu method, and Coomassie brilliant blue method, respectively. The component profiling of DHWE samples was analyzed using an ultra-performance liquid chromatography (UPLC)–Orbitrap–mass spectrometry (MS) system (UPLC, Vanquish, Thermo Fisher Scientific, Waltham, MA, USA; MS, HFX, Thermo Fisher Scientific, Waltham, MA, USA). The results showed that the contents of carbohydrates, total polyphenols, and proteins in DHWE were 82.93 ± 1.10%, 0.51 ± 0.03%, and 0.38 ± 0.06%, respectively. With respect to the metabolite composition of DHWE, the peak area ratios of different types of metabolites were as follows: phenylpropanoids and polyketides (39.30%), lipids and lipid-like molecules (10.88%), nucleosides, nucleotides, and analogs (10.54%), organic oxygen compounds (10.48%), organic acids and derivatives (9.25%), organoheterocyclic compounds (6.90%), unclassified metabolites (6.62%), benzenoids (3.72%), lignans, neolignans, and related compounds (0.72%), alkaloids and derivatives (0.69%), hydrocarbon derivatives (0.01%), and organic nitrogen compounds (0.003%).

### 4.2. Animal Experimental Design

The source of experimental animals, the establishment of T1D models and the experimental procedure were described in our published paper [13]. Briefly, rats (weight = 200 ± 20 g) were induced the STZ-T1D model via the intraperitoneal injection of streptozotocin at a dose of 60 mg/kg/body weight after fasting for 12 h. Rats with fasting blood glucose levels > 16.7 mmol/L and symptoms of polydipsia and polyuria 72 h later were considered successful STZ-T1D models. 24 STZ-T1D rats were randomly divided into three groups: the T1D model group (MOD group), the DHWE-treated T1D model group (DHWE group), and the metformin (positive drug)-treated T1D model group (MET group), with 8 rats in each group. In addition, 8 healthy rats from the same batch were used as the normal control group (CON group). Rats in the CON and MOD groups were given distilled water (1 mL/100 g·BW) by gavage every morning. Rats in the DHWE and MET groups received DHWE (1300 mg/kg·BW) or metformin (100 mg/kg·BW) by gavage every morning, respectively. The dosage was adjusted weekly according to body weight changes. The experiment lasted for 28 days.

### 4.3. Fecal Sample Collection

On the morning of the 28th day, after 12 h of fasting, rats were euthanized by intraperitoneal injection of a lethal dose of sodium pentobarbital (200 mg/kg·BW). The abdominal cavity was opened, and fecal pellets in the rectum were collected with sterile forceps into cryogenic vials. The samples were immediately frozen in liquid nitrogen and stored at −80 °C until analysis.

### 4.4. Metagenomic Analysis of Gut Microbiota

#### 4.4.1. DNA Extraction and PCR Amplification

DNA was extracted from fecal samples using the cetyltrimethylammonium bromide (CTAB) method (see Supplementary Material S1). DNA quality and fragment size were analyzed using an Agilent 5400 Fragment Analyzer System (Agilent Technologies, Inc., Santa Clara, CA, USA; manufactured in Malaysia). This system is based on parallel capillary electrophoresis, allowing automated high-throughput quality control, sizing, and quantitation of nucleic acid samples.

#### 4.4.2. Library Construction and Inspection

Sequencing libraries were generated using the NEBNext^®^ Ultra^TM^ DNA Library Prep Kit for Illumina (NEB, Ipswich, MA, USA) following the manufacturer’s instructions, and index codes were added to attribute sequences to each sample. Briefly, DNA samples were fragmented by sonication to 350 bp, followed by end polishing, A-tailing, ligation with full-length Illumina sequencing adaptors, and further PCR amplification. Finally, PCR products were purified using the AMPure XP system. Library size distribution was assessed using an Agilent 2100 Bioanalyzer (Agilent Technologies, Santa Clara, CA, USA), and library quantification was performed by real-time PCR.

#### 4.4.3. Sequencing

Index-coded samples were clustered on a cBot Cluster Generation System using the Illumina PE Cluster Kit (Illumina, San Diego, CA, USA) according to the manufacturer’s instructions. After cluster generation, the library preparations were sequenced on the Illumina Novaseq 6000 platform, and 150 bp paired-end reads were generated.

#### 4.4.4. Bioinformatics Analysis

##### Data Quality Control and Host Sequence Removal

Raw sequencing data of bacteria, fungi and viruses from fecal samples were generated via metagenomic sequencing on the Illumina NovaSeq high-throughput platform. To ensure the accuracy and reliability of downstream analyses, raw reads were preprocessed using Kneaddata incorporating Trimmomatic v0.39 and Bowtie2 v2.3.5.1, with detailed procedures as follows: (1) Quality control was performed using Trimmomatic to remove sequencing adapters (parameter: ILLUMINACLIP:adapters.fa:2:30:10) and low-quality reads. A 4 bp sliding-window strategy was adopted, where bases at the 3′-end were trimmed when the average Phred quality score fell below 20 (corresponding to 99% base-calling accuracy; parameter: SLIDINGWINDOW:4:20). Reads shorter than 50 bp were further discarded (parameter: MINLEN:50). (2) Host-derived contamination was eliminated by aligning clean reads against the host genome database using Bowtie2 with the parameter very-sensitive, followed by filtering out host-mapped reads. (3) FastQC v0.11.9 was applied to assess the efficiency and reliability of quality control [56,57,58].

##### Taxonomic Annotation

Kraken2 combined with a self-build microbial database (including bacterial, fungal, archaeal and viral sequences screened from the NCBI NT nucleic acid database and RefSeq whole-genome database) was used to identify species in each samples. Bracken was then applied to estimate the actual relative abundance of each species. Briefly, Kraken2 is a K-mer-based classification tool. The local Kraken2 database used in this study contained 16,799 published bacterial genomes [59,60,61,62].

##### Functional Annotation Based on Clean Reads

Clean reads after quality control and host removal were aligned against the UniRef90 database using HUMAnN3 v3.6 software (based on DIAMOND). Functional annotation and relative abundance profiles were generated according to the correspondence between UniRef90 IDs and functional database [63,64,65,66].

Based on species and functional abundance profiles, the following analyses were performed: abundance clustering analysis, principal coordinates analysis (PCoA), sample clustering, and linear discriminant analysis effect size (LEfse) to reveal differences in microbial community structure and functional composition among groups. With grouping information, LEfSe biomarker identification and Dunn’s test were conducted to explore differentially abundant taxa and functions between groups [67]. Differences in the abundance of functional pathways were compared using Specifications Trust Association and Marker Gene Profile (STAMP v2.1.3) software.

##### Correlations of Gut Microbiota with Serum Biochemical Indicators

Spearman correlation analysis was performed to evaluate the relationship between blood biochemical indices and gut microbiota composition. The blood biochemical indices used in this study have been reported in our previous publication [13].

##### Joint Analysis of Gut Microbiota Metagenome and Plasma Metabolome

Integrated analysis of metagenomics and metabolomics was performed to explore the associations between microbial characteristics and metabolite profiles using correlation analysis. This approach helps identify key microorganisms that are significantly correlated with metabolite levels, thereby improving understanding of the interactions between the gut microbiome and plasma metabolome in physiological and pathological processes. The effects of DHWE and MET on the plasma metabolome of STZ-T1D rats have been reported in our previous study [13]. In the present study, spearman correlation analysis was used to generate a correlation heatmap between the top 20 microbial features and the top 25 metabolomic features.

## 5. Conclusions

Using gut metagenomic analysis, this study provided new insights into gut microbiota alterations following DHWE intervention in STZ-T1D rats. Our results demonstrated that DHWE significantly increased the abundance of *Adlercreutzia*, *Adlercreutzia equolifaciens* and reduced the abundance of *Megamonas*, *Megamonas funiformis* in STZ-T1D rats. Moreover, DHWE enriched numerous microbial metabolic pathways in the gut of T1D rats, whereas the MET group only enriched two metabolic pathways. Nevertheless, both DHWE and MET were able to remodel the gut microbiota of STZ-T1D rats towards a healthy status and alleviate the progression of T1D. Future studies based on these findings are warranted to further elucidate the mechanisms underlying the roles of key bacterial species in the initiation and progression of T1D, which may provide novel strategies for the more effective control of T1D.

## Figures and Tables

**Table 1:** Differences in α-diversity indices of gut microbiota among experimental groups of T1D rats.

Group	Simpson	Shannon	Coverage	Chao1	ACE
CON	0.87 ± 0.06	4.02 ± 0.72 ^ab^	0.99998 ± 0.000006	588.9 ± 91.4	580.8 ± 82.4 ^a^
MOD	0.85 ± 0.09	4.03 ± 0.57 ^ab^	0.99999 ± 0.000009	541.7 ± 64.0	535.2 ± 62.9 ^ab^
DHWE	0.88 ± 0.07	4.42 ± 0.60 ^a^	0.99998 ± 0.000008	504.5 ± 56.7	495.6 ± 57.8 ^b^
MET	0.81 ± 0.09	3.66 ± 0.70 ^b^	0.99999 ± 0.000005	510.3 ± 82.1	505.2 ± 75.1 ^b^

Note: The α-diversity indices were compared among groups by one-way analysis of variance (ANOVA), followed by Duncan’s multiple range test for post hoc comparisons. Different lowercase superscript letters in the same column indicate statistically significant differences (*p* < 0.05), whereas the same letters indicate no significant differences. Data are expressed as mean ± SD.

**Table 2:** Comparison of the relative abundance (%) of dominant flora composition at phylum, genus and species level, respectively.

Group	CON Group	MOD Group	DHWE Group	MET Group
Phylum name				
*Actinomycetota*	15.81 ± 4.68	9.34 ± 6.67	15.25 ± 8.13	12.15 ± 8.63
*Bacillota*	58.52 ± 16.92	32.45 ± 16.19	53.26 ± 18.78	51.01 ± 26.26
*Bacteroidota*	16.55 ± 11.66	40.14 ± 22.94	15.73 ± 15.61	19.52 ± 27.43
*Bacteroidota*: *Bacillota*	0.35 ± 0.28	1.75 ± 1.44 *	0.38 ± 0.42 ^#^	1.16 ± 2.47 **
genus name				
*Bifidobacterium*	0.53 ± 0.46	6.50 ± 6.0	2.97 ± 4.91	2.15 ± 4.73
*Adlercreutzia*	13.65 ± 4.50	1.98 ± 1.62 ***	10.75 ± 4.09 ^##^	8.42 ± 8.21 *
*Limosilactobacillus*	11.48 ± 5.58	2.21 ± 1.93 ***	4.49 ± 2.68 *	6.42 ± 4.83 ^#^
*Ruminococcus*	0.26 ± 0.61	0.49 ± 0.29	1.33 ± 0.79 **	0.81 ± 1.22
*Romboutsia*	7.16 ± 4.44	0.83 ± 1.23 ***	2.32 ± 2.0 **	1.16 ± 0.87 **
*Megamonas*	0.00 ± 0.00	2.74 ± 2.68 ***	0.60 ± 1.55 ^##^	0.51 ± 0.80 ^#^
*Prevotella*	0.04 ± 0.05	15.23 ± 19.55 ***	0.71 ± 1.09 *	7.74 ± 20.39 ^##^
*Klebsiella*	0.07 ± 0.09	2.45 ± 3.69 **	0.87 ± 1.57 *	1.14 ± 1.55 **
Species name				
*Bifidobacterium_pseudolongum*	0.54 ± 0.48	5.73 ± 5.23	3.23 ± 5.45	2.51 ± 5.79
*Adlercreutzia_equolifaciens*	14.04 ± 4.34	2.09 ± 1.70 ***	10.69 ± 4.73 ^##^	8.63 ± 8.30 *
*Lactobacillus_johnsonii*	8.34 ± 4.91	6.21 ± 3.10	11.13 ± 5.17	16.77 ± 14.12
*Limosilactobacillus_reuteri*	11.76 ± 5.29	2.25 ± 2.06 ***	4.57 ± 2.31 *	6.51 ± 4.56 ^#^
*Faecalibacterium_prausnitzii*	0.33 ± 0.77	0.42 ± 0.27	1.47 ± 1.56	0.70 ± 0.71
*Ruminococcus*_sp._SR1/5	0.24 ± 0.58	0.33 ± 0.19	1.07 ± 0.54 **	0.73 ± 1.20
*Romboutsia_ilealis*	7.58 ± 4.27	0.90 ± 1.37 ***	2.26 ± 2.29 **	1.27 ± 1.02 **
*Megamonas_funiformis*	0.00 ± 0.00	1.84 ± 1.66 ***	0.41 ± 1.06 ^##^	0.35 ± 0.52 ^#^
*Bacteroides_uniformis*	0.50 ± 0.59	1.92 ± 2.68	0.23 ± 0.22	0.26 ± 0.29
*Prevotella_copri*	0.01 ± 0.01	15.94 ± 20.54 ***	1.00 ± 1.34 *	8.08 ± 21.32 ^#^
*Klebsiella_pneumoniae*	0.07 ± 0.09	2.37 ± 3.54 **	0.85 ± 1.57 *	0.71 ± 0.72 **

Note: The relative abundance (%) of the dominant gut microbiota at phylum, genus and species levels was analyzed. Due to the skewed distribution of microbial abundance data, non-parametric Kruskal–Wallis H-test followed by Dunn’s multiple comparisons was used for intergroup statistical comparison. Mean ± standard deviation was presented merely for intuitive data visualization rather than parametric statistical inference. * *p* < 0.05, ** *p* < 0.01, *** *p* < 0.001 vs. control (CON) group; ^#^ *p* < 0.05, ^##^ *p* < 0.01 vs. model (MOD) group. The original data of relative abundance and statistical results are provided in Tables S2–S4.

## Data Availability

The original contributions presented in this study are included in the article/Supplementary Materials. Further inquiries can be directed to the corresponding authors.

## Supplementary Materials

The following supporting information can be downloaded at: https://www.mdpi.com/article/10.3390/ijms27125308/s1.

## Abbreviations

ABC	ATP-binding cassette
ACar	Acylcarnitine
ACE	adjusted richness estimator
APN-AMPK-PPARα	adiponectin-adenosine 5′-monophosphate-activated protein kinase-peroxisome proliferator-activated receptor α
CRP	C-reactive protein
CTAB	cetyltrimethylammonium bromide
DHWE	*Dendrobium huoshanense* water extract
FAHFA	acid esters of hydroxy fatty acids
FDR	false discovery rate
FC	fold change
GSEA	gene set enrichment analysis
GO	Gene Ontology
KEGG	Kyoto Encyclopedia of Genes and Genomes
KW	Kruskal–Wallis test
LDA	linear discriminant analysis
LEfse	linear discriminant analysis effect size
LPC	lysophosphatidylcholine
MAFLD	metabolic dysfunction-associated fatty liver disease
MDA	malondialdehyde
MET	metformin
ORA	over-representation analysis
PC	phosphatidylcholine
PCoA	principal coordinates analysis
PCOS-IR	polycystic ovary syndrome-insulin resistance
PCOS-NIR	PCOS-non-insulin-resistant
PTS	phosphotransferase system
STAMP	Specifications Trust Association and Marker Gene Profile
STZ-T1D	streptozotocin-induced type 1 diabetic
T-AOC	total antioxidant capacity
Th1	T helper 1
Th17	T helper 17
TLR2	toll-like receptor 2
Treg cell	regulatory T cell
