# Supplementary material for: Metagenomic Insights into Gut Microbiota Alterations Following Dendrobium huoshanense Water Extract Intervention in Streptozotocin-Induced Type 1 Diabetic Rats
Source: Int J Mol Sci. 2026 Jun 11;27(12):5308. doi: 10.3390/ijms27125308 (PMC13299921; doi:10.3390/ijms27125308)
Supplement: Supplementary file 1 [file ijms-27-05308-s001.zip › Fig. S1.pdf]

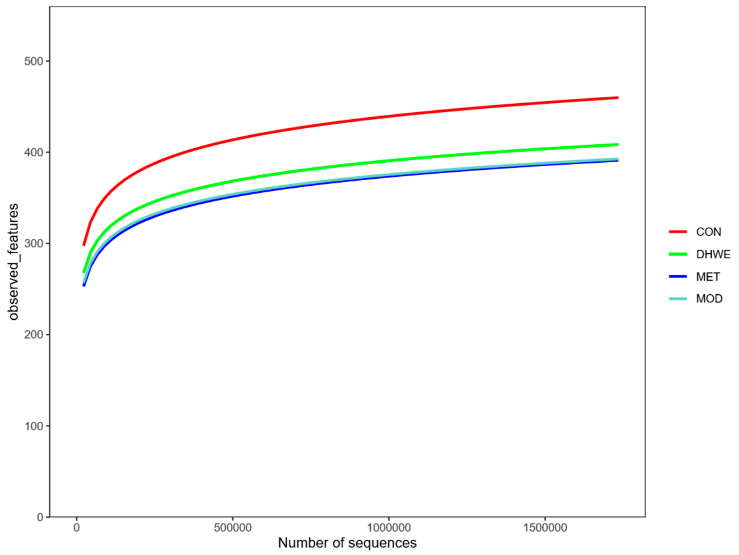

Figure S1. Rarefaction curve of observed features across experimental groups. The curves for all groups (CON, DHWE, MET, MOD) reached a plateau when the sequencing depth reached 1,500,000 sequences, indicating that the sequencing depth was sufficient to capture the majority of microbial diversity in the samples.
