# Supplementary material for: Metagenomic Insights into Gut Microbiota Alterations Following Dendrobium huoshanense Water Extract Intervention in Streptozotocin-Induced Type 1 Diabetic Rats
Source: Int J Mol Sci. 2026 Jun 11;27(12):5308. doi: 10.3390/ijms27125308 (PMC13299921; doi:10.3390/ijms27125308)
Supplement: Supplementary file 1 [file ijms-27-05308-s001.zip › Fig. S2.pdf]

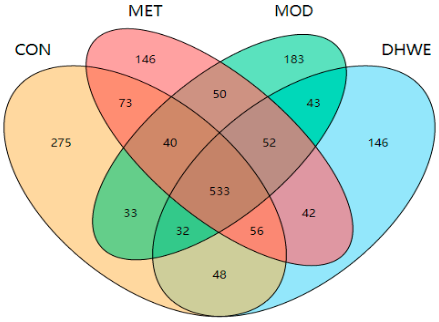

Figure S2**.** Venn diagram of bacterial taxa.The Venn diagram shows the number of common and unique bacterial taxa among groups. Each ellipse represents one group.
