# Supplementary material for: Metagenomic Insights into Gut Microbiota Alterations Following Dendrobium huoshanense Water Extract Intervention in Streptozotocin-Induced Type 1 Diabetic Rats
Source: Int J Mol Sci. 2026 Jun 11;27(12):5308. doi: 10.3390/ijms27125308 (PMC13299921; doi:10.3390/ijms27125308)
Supplement: Supplementary file 1 [file ijms-27-05308-s001.zip › Fig. S4.pdf]

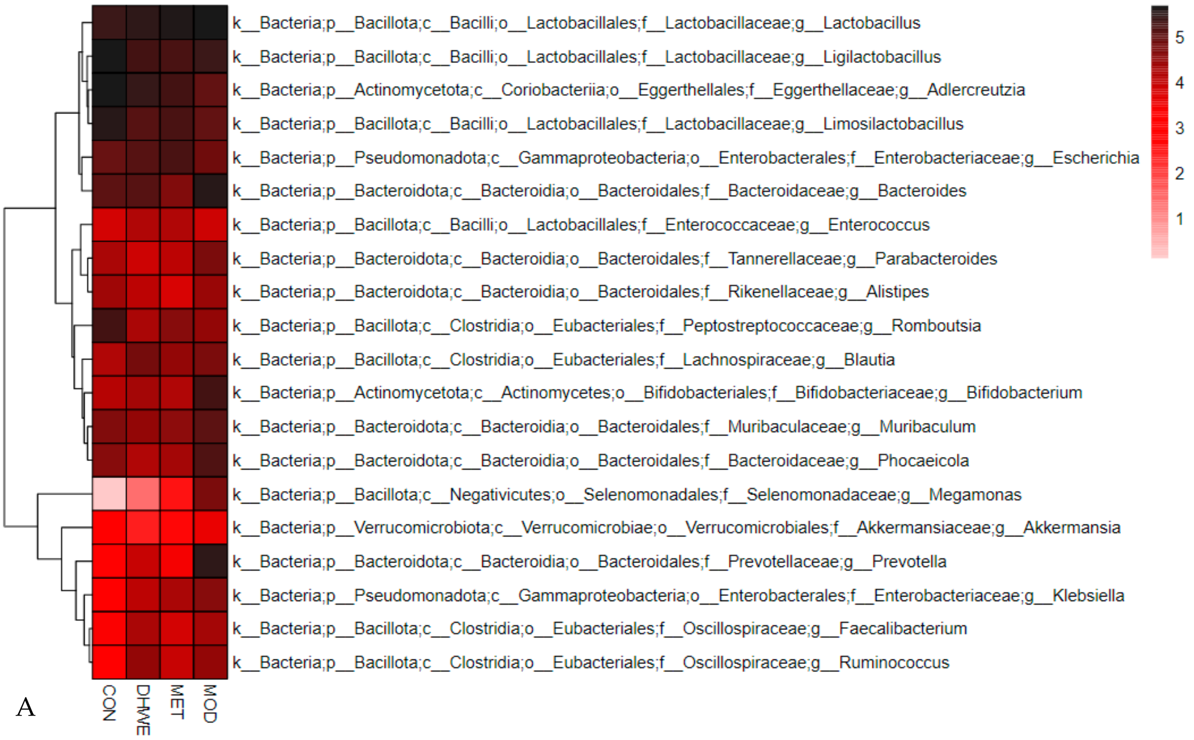

A

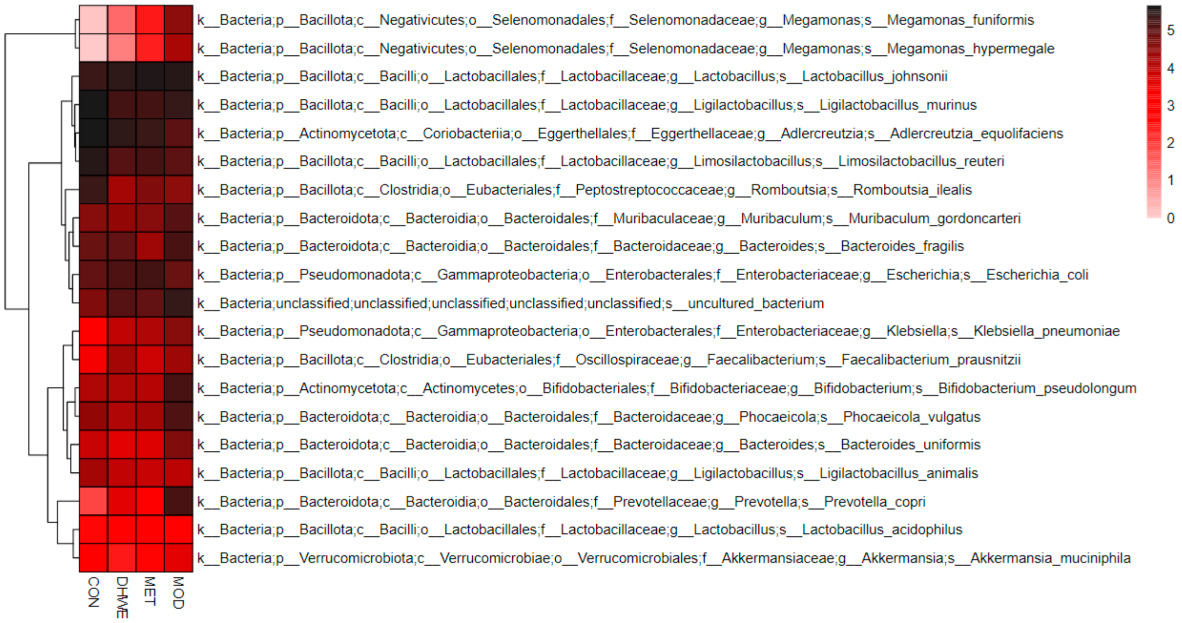

B

Figure S4. Heatmap of the top 20 most abundant bacteria at the genus and species levels, with hierarchical clustering. (A) Genus level; (B) Species level. Legend: Rows represent bacterial taxa, annotated at the genus (A) or species (B) level. Columns represent experimental groups: CON (control), DHME (treatment 1), MET (treatment 2), and MOD (model). The color intensity (ranging from black to red) indicates the relative abundance of each taxon, with black representing low abundance and red representing high abundance. Hierarchical clustering dendrograms on the left and top show the clustering of

bacterial taxa and samples, respectively, based on the similarity of their microbial community profiles.
