# Supplementary material for: Metagenomic Insights into Gut Microbiota Alterations Following Dendrobium huoshanense Water Extract Intervention in Streptozotocin-Induced Type 1 Diabetic Rats
Source: Int J Mol Sci. 2026 Jun 11;27(12):5308. doi: 10.3390/ijms27125308 (PMC13299921; doi:10.3390/ijms27125308)
Supplement: Supplementary file 1 [file ijms-27-05308-s001.zip › Supplementary material S1.pdf]

Supplementary material S1

Genomic DNA was extracted from fecal samples using the cetyltrimethylammonium bromide (CTAB) method. Pipette 1000 μL of CTAB lysis buffer into a 2.0 mL EP tube, add lysozyme, and then add an appropriate amount of fecal sample to the lysis buffer. Incubate in a 65 ℃ water bath, inverting and mixing several times during the process to fully separate and lyse the sample. After centrifugation, take the supernatant, add phenol (pH 8.0):chloroform:isoamyl alcohol (25:24:1), invert and mix thoroughly, then centrifuge at 12000 rpm for 10 minutes. Take the resulting supernatant, add chloroform:isoamyl alcohol (24:1), invert and mix again, and centrifuge at 12000 rpm for 10 minutes. Transfer the supernatant into a 1.5 mL centrifuge tube, add isopropanol, shake the tube up and down, and allow the DNA to precipitate at -20℃. Centrifuge at 12000 rpm for 10 minutes. Carefully pour out the liquid, taking care not to pour out the precipitate. Wash the precipitate twice with 1 mL of 75% ethanol. Collect the remaining small amount of liquid by centrifuging again, and then aspirate it with a pipette tip. Dry the precipitate on an ultra - clean workbench at room temperature. Add ddH_2O to dissolve the DNA sample. Incubate at 60℃ for 10 minutes to facilitate dissolution. Add 1μL of RNase A to digest RNA and let it stand at 37℃ for 15 minutes. Finally, detect the concentration, integrity, and purity of the DNA.
