# Supplementary material for: Metagenomic Insights into Gut Microbiota Alterations Following Dendrobium huoshanense Water Extract Intervention in Streptozotocin-Induced Type 1 Diabetic Rats
Source: Int J Mol Sci. 2026 Jun 11;27(12):5308. doi: 10.3390/ijms27125308 (PMC13299921; doi:10.3390/ijms27125308)
Supplement: Supplementary file 1 [file ijms-27-05308-s001.zip › Table S1.pdf]

Table S1. Differences in KEGG pathway abundance among groups.

	Feature	KW_p value	DunnTest_comparsion	Dunntest_Z	DunnTest_ value
KEGG level1	Metabolism	0.0304	CON-MOD	-2.8493	0.0022
		0.0304	DHWE-MOD	-2.1360	0.0163
		0.0304	MET-MOD	-2.0948	0.0181
KEGG level2	Amino acid metabolism	0.0423	CON - MOD	-2.1203	0.0170
		0.0423	DHWE - MOD	-1.8186	0.0345
		0.0423	MET - MOD	-2.7323	0.0031
	Glycan biosynthesis and metabolism	0.0083	CON - MOD	-2.0263	0.0214
		0.0083	DHWE - MOD	-2.9591	0.0015
		0.0083	MET - MOD	-3.0055	0.0013
	Transport and catabolism	0.0285	DHWE - MOD	-2.5828	0.0049
		0.0285	MET - MOD	-2.5805	0.0049
		0.0285	CON - MOD	-2.1987	0.0139
KEGG level3	Arginine biosynthesis	0.0288	MET - MOD	-2.1555	0.0156
	Cysteine and methionine metabolism	0.0478	MET - MOD	-2.3680	0.0089
		0.0478	CON - MOD	-1.8029	0.0357
	Phenylalanine, tyrosine and tryptophan biosynthesis	0.0076	CON - MOD	-3.1825	0.0007
		0.0076	MET - MOD	-2.7930	0.0026
		0.0076	DHWE - MOD	-2.1125	0.0173
	Ether lipid metabolism	0.0166	DHWE - MOD	1.9168	0.0276
	Sphingolipid metabolism	0.0121	DHWE - MOD	-2.9944	0.0014
		0.0121	MET - MOD	-2.7019	0.0034
	Fructose and mannose metabolism	0.0327	MET - MOD	1.7305	0.0418
	Galactose metabolism	0.0373	DHWE - MOD	1.8146	0.0348
	Various types of N-glycan biosynthesis	0.0478	MET - MOD	-2.4894	0.0064
		0.0478	DHWE - MOD	-1.6971	0.0448
	Other glycan degradation	0.0146	DHWE - MOD	-2.5907	0.0048
		0.0146	MET - MOD	-3.0055	0.0013
		0.0146	CON - MOD	-2.0420	0.0206
	Phosphotransferase system (PTS)	0.0453	MET - MOD	2.5805	0.0049
	Atrazine degradation	0.0049	CON - MOD	3.1433	0.0008
		0.0049	MET - MOD	1.7001	0.0446
	Vitamin B_6 metabolism	0.0292	CON - MOD	-2.9473	0.0016
		0.0292	MET - MOD	-2.0037	0.0226
		0.0292	DHWE - MOD	-1.7676	0.0386
	Vitamin B_2 metabolism	0.0456	DHWE - MOD	-1.8538	0.0319
	Vitamin B_1 metabolism	0.0219	CON - MOD	2.8572	0.0021
	Biosynthesis of ansamycins	0.0136	DHWE - MOD	2.2732	0.0115
	Carbapenem biosynthesis	0.0125	DHWE - MOD	-1.7049	0.0441
	Novobiocin biosynthesis	0.0129	CON - MOD	-2.9042	0.0018
		0.0129	MET - MOD	-2.7627	0.0029
		0.0129	DHWE - MOD	-2.3006	0.0107
	Streptomycin biosynthesis	0.0157	DHWE - MOD	2.1870	0.0144
	Tetracycline biosynthesis	0.0017	DHWE - MOD	-3.8635	0.0001
		0.0017	MET - MOD	-2.3434	0.0096
		0.0017	CON - MOD	-2.1750	0.0148

Note: The relative abundance of KEGG pathways among groups was analyzed using the Kruskal-Wallis (KW) test, followed by Dunn's multiple comparison test for post-hoc pairwise comparisons. A p-value < 0.05 was considered statistically significant. Only data showing statistically significant differences compared to the MOD group (KW $p < 0.05$ and Dunn test $p < 0.05$) are displayed in this table.
