# Supplementary figures and images for: Metagenomic Insights into Gut Microbiota Alterations Following Dendrobium huoshanense Water Extract Intervention in Streptozotocin-Induced Type 1 Diabetic Rats
Source: Int J Mol Sci. 2026 Jun 11;27(12):5308. doi: 10.3390/ijms27125308 (PMC13299921; doi:10.3390/ijms27125308)

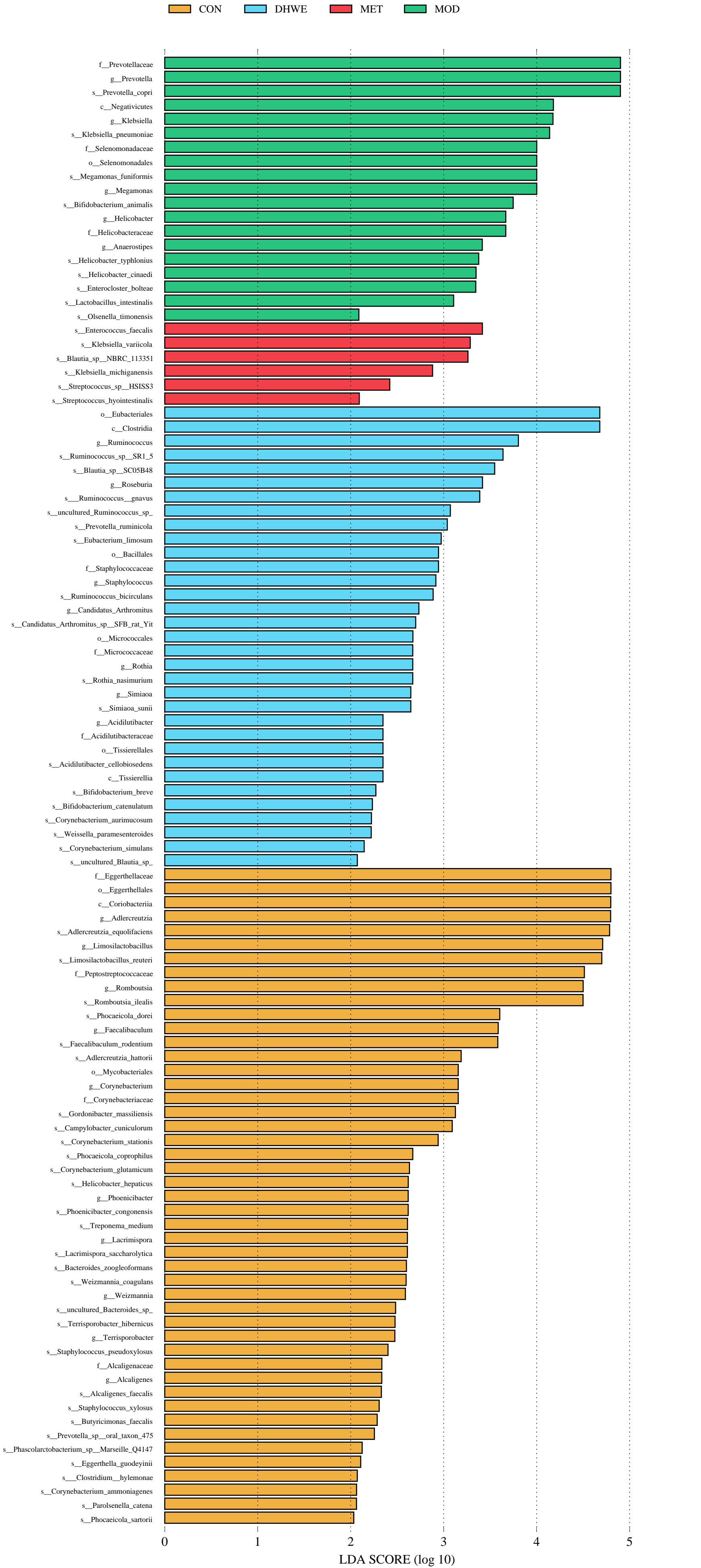

Supplement: Supplementary file 1 [file ijms-27-05308-s001.zip › Fig. S3-comparison using Lefse LDA2.pdf]
